# Monitoring the Microwave Synthesis of *d* ^0^‐Free Disordered Rocksalt Cathodes Using In Situ Infrared Pyrometry

**DOI:** 10.1002/anie.1884534

**Published:** 2026-08-05

**Authors:** Erick A. Lawrence, Matthew A. Wright, Tianyu Li, Euan N. Bassey, Vijay Kumar, Shiyu Yuan, Yangying Zhu, Pierre‐Etienne Cabelguen, Raphaële J. Clément

**Affiliations:** ^1^ Materials Department and Materials Research Laboratory University of California Santa Barbara Santa Barbara California USA; ^2^ Present address: Department of Chemistry Louisiana Tech University, Ruston Los Angles USA; ^3^ Present address: Centre de RMN à Très Hauts Champs de Lyon (UMR 5082 CNRS, ENS Lyon, UCB Lyon 1) Université de Lyon Villeurbanne France; ^4^ Department of Mechanical Engineering University of California Santa Barbara Santa Barbara California USA; ^5^ Umicore New Business Incubation Brussels Belgium; ^6^ Max Planck Institute for Solid State Research Stuttgart Germany

**Keywords:** disordered rocksalt, in situ infrared monitoring, Li‐ion batteries, microwave chemistry, solid‐state reactions

## Abstract

A detailed understanding of solid‐state reaction pathways is essential for connecting predictive frameworks, such as density functional theory and machine learning, with experimental synthesis. Microwave synthesis has emerged as a powerful route for preparing inorganic materials, yet the mechanisms governing microwave‐driven processes remain poorly understood, particularly for metastable compounds whose formation is highly sensitive to synthesis conditions. Disordered rocksalt oxides (DRX) are high‐temperature metastable phases of interest as next‐generation Li‐ion cathodes. Here, we investigate the microwave reaction pathway of ${\mathrm{Li}}_{1.1}{\mathrm{Mn}}_{0.9}O_{1.9}F_{0.1}$. Combining ex situ phase identification using x‐ray diffraction and solid‐state NMR with in situ infrared thermography, we show that the reaction proceeds through a reentrant order–disorder–order transformation. Layered Li‐Mn‐O intermediates disorder above 945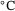 to form the DRX phase, while continued heating drives reordering back to layered structures. Infrared profiles reveal a distinct feature marking completion of the disordering transition, enabling precise reaction termination to maximize DRX phase purity. We further examine the impact of phase purity on the “δ‐phase” transition during electrochemical cycling and find that residual layered phases minimally affect performance. These findings indicate that ${\mathrm{Li}}_{1.1}{\mathrm{Mn}}_{0.9}O_{1.9}F_{0.1}$ is only stable near 945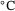, yet its electrochemical performance tolerates synthesis‐induced impurities.

## Graphical abstract

Despite its promise for producing metastable materials, the mechanisms underlying solid‐state microwave synthesis remain poorly understood. By combining in situ thermography with x‐ray diffraction and solid‐state NMR, this study uncovers a surprising order–disorder–order reaction pathway during the microwave synthesis of the ${\mathrm{Li}}_{1.1}{\mathrm{Mn}}_{0.9}O_{1.9}F_{0.1}$ cathode, identifies the narrow temperature range that stabilizes the disordered rocksalt phase, and reveals an unexpected tolerance to synthesis‐induced impurities.

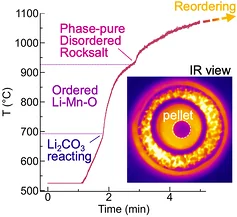

## 1 Introduction

Microwave heating, first adopted by organic chemists [1, 2, 3], has since become a widely used method to drive both solution‐state [4, 5] and solid‐state reactions, enabling the preparation of diverse materials, including oxides [6, 7, 8, 9], chalcogenides [10, 11, 12, 13], borides [14, 15], carbides [16, 17], silicides [18, 19], and nitrides [20, 21]. Beyond its versatility, microwave heating offers a unique synthetic route capable of stabilizing phases that are challenging to obtain through conventional heating [22]. As density functional theory and machine learning methods have become increasingly powerful tools for predicting stable and metastable phases and their synthetic pathways, real‐time in situ monitoring of complex reactions has emerged as essential for bridging these computational predictions with experimentally accessible synthesis routes.

For solution‐state microwave‐assisted reactions, in situ techniques are routinely applied by virtue of low heating rates and reaction temperatures; Raman [23, 24, 25], infrared (IR) [26], and nuclear magnetic resonance (NMR) [27] spectroscopies have all been successfully employed to elucidate reaction mechanisms. The microwave‐assisted synthesis of solid materials has been studied primarily for systems that crystallize from solution during hydrothermal synthesis (e.g., various zeolite and iron oxide nanoparticles), where in situ scattering techniques have been successfully employed to probe reaction mechanisms [28, 29, 30, 31].

Solid‐state microwave‐assisted reactions are more challenging to probe in situ due to the far higher heating rates and reaction temperatures. Accordingly, most in situ solid‐state microwave studies have used scattering methods to investigate either the effects of microwave irradiation on the structural parameters of pure compounds (e.g., lattice parameters, atomic displacement parameters, and bond angles) [32, 33], or on simple thermally driven phase transitions between stable polymorphs, such as the α to β transition in AgI or the anatase to rutile transition in ${\mathrm{TiO}}_{2}$ [34, 35]. As noted by Kitchen et al. [4], a central challenge in the in situ investigation of solid‐state microwave synthesis is the rapid heating, on the order of hundreds of degrees per minute, that can drive reactions on timescales that demand high temporal resolution for meaningful in situ characterization. As a result, in situ monitoring has frequently been limited to surface temperature measurements using infrared (IR) pyrometry or optical fiber thermometry [36, 37, 38]. A notable exception is the recent work of Jamboretz et al., who employed in situ Raman spectroscopy to track the reaction pathway during the microwave synthesis of layered ${\mathrm{Na}}_{x}{\mathrm{CoO}}_{2}$ cathode materials [7].

Despite the known limitations of IR pyrometry (particularly, its strong dependence on emissivity, which can vary substantially over the course of a reaction) [4, 38], several studies of microwave and other ultrafast synthesis methods have reported distinct features in the recorded temperature profiles [7, 39]. While these features are sometimes correlated with phase transitions [39], their underlying chemical origins have not been systematically investigated.

Disordered rocksalt oxides (DRX) are a promising class of lithium‐ion battery cathode materials due to their high energy density and compositional flexibility using earth‐abundant transition metals. These materials are considered high‐temperature metastable phases because Li and the redox‐active transition metal (e.g., Mn, Fe, V, Cr) share the same crystallographic sites. This cation disorder introduces significant lattice strain arising from the mismatch in ionic radii between the species (exceeding 15% in the case of ${\mathrm{Li}}^{+}$ and ${\mathrm{Mn}}^{3+}$ when they occupy the same lattice positions. The incorporation of $d^{0}$ cations such as ${\mathrm{Ti}}^{4+}$, ${\mathrm{Zr}}^{4+}$, ${\mathrm{Nb}}^{5+}$, or ${\mathrm{Mo}}^{6+}$ can stabilize the DRX structure because their electronic band energies are largely insensitive to substantial octahedral distortions [40]. As a result, these species can accommodate local site distortions in mixed transition metal compositions, helping to relieve lattice strain. However, this stabilization comes at the expense of electrochemical capacity, as increasing the $d^{0}$ content reduces the overall redox activity of the transition metal sublattice [41]. Therefore, recent research has focused on minimizing or eliminating $d^{0}$ species, culminating in a recent report of the synthesis of several $d^{0}$‐free DRX materials [42]. This was achieved through rapid microwave heating followed by thermal quenching in water, which kinetically traps the high‐temperature, $d^{0}$‐free DRX phase. For example, substituting redox‐inactive ${\mathrm{Ti}}^{4+}$ in ${\mathrm{Li}}_{1.1}\mathrm{Mn}$(III)
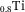
(IV)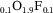 DRX (LMT81) with redox‐active ${\mathrm{Mn}}^{4+}$ to form the $d^{0}$‐free ${\mathrm{Li}}_{1.1}\mathrm{Mn}$(III)
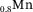
(IV)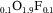 DRX (LM9) yields a highly metastable DRX phase with an expanded transition metal redox reservoir.

In this work, we investigate the microwave synthesis of the $d^{0}$‐free LM9 DRX. We elucidate the microwave reaction pathway and identify a reentrant transformation mechanism: the precursors initially react to form layered Li–Mn–O intermediate phases, which disorder into the DRX structure above 945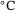; upon continued heating, however, the system re‐orders, reverting to layered phases. The reaction pathway is monitored using a combination of in situ IR thermal imaging and ex situ phase analysis. Structural evolution is characterized by Rietveld refinement against high‐resolution synchrotron x‐ray diffraction (sXRD) data and 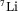 solid‐state nuclear magnetic resonance (NMR) spectroscopy. We correlate the observed phase evolution with two distinct features in the temperature profile obtained from the in situ IR measurements and unambiguously determine their chemical origins. Finally, we demonstrate that the electrochemical performance of LM9 is largely insensitive to both the reentrant phase formation and the increased particle size resulting from prolonged microwave heating.

## 2 Results and Discussion

### 2.1 In Situ and Ex Situ Characterization of the LM9 Reaction Pathway

Microwave synthesis of DRX materials [42, 43] uses a double‐crucible configuration in which a pellet of precursor powders is placed in a small alumina inner crucible. This inner crucible is embedded within a bed of activated charcoal contained in a larger outer crucible (Figure 1a–c). The activated charcoal acts as a microwave susceptor, absorbing microwave radiation and converting it to heat. The heat is conducted through the base of the inner crucible to the precursor pellet, thereby driving the chemical reaction. Using this approach, DRX compounds have been obtained with high phase purity after only 5 min of microwave heating in air, followed by rapid quenching in water and subsequent drying of the pellet on a hot plate to preserve the high‐temperature disordered phase [42, 43, 44]. The same microwave synthesis process in air was adopted for this work, and the overall synthesis workflow is schematically illustrated in Figure 1a. Additional discussion of the roles of the charcoal susceptor and air atmosphere in the formation of surface impurity phases is provided in Note S1.

**FIGURE 1:**
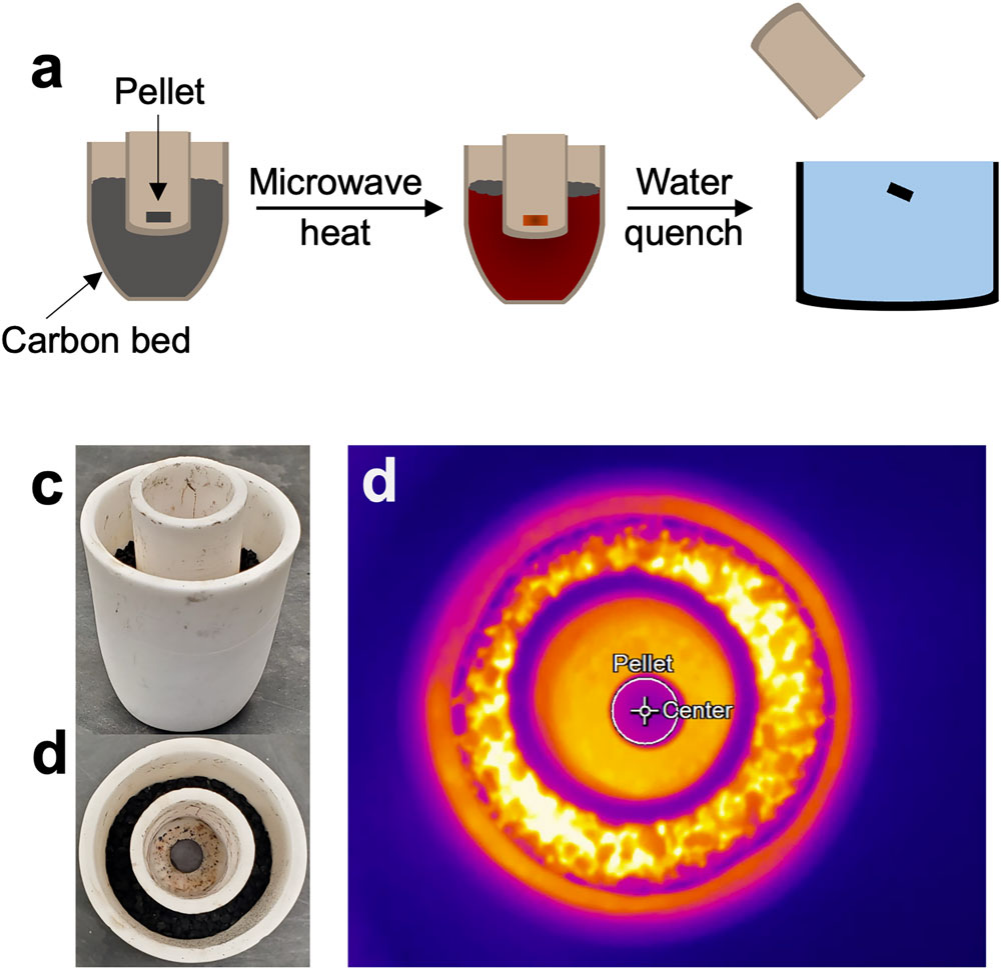
(a) Schematic representation of the microwave DRX synthesis workflow. The precursor pellet is shown as the central circle within the inner crucible. (b) Side‐on and (c) top‐down view of the double‐crucible configuration.(d) Representative screenshot from the IR camera during microwave synthesis at 1200 W. The reaction is viewed from a top‐down perspective, corresponding to the orientation shown in (c).

Previous reports of microwave DRX synthesis employed repurposed commercial kitchen microwaves [42, 43, 44]. To better understand the microwave reaction mechanism, we instead use a customized Milestone microwave reactor equipped with a viewport that enables in situ surface temperature measurements of the LM9 precursor pellet using an IR camera. The IR camera records the reaction temperature from a top‐down perspective (Figure 1d). An IR thermal imaging video of a 5‐min LM9 synthesis conducted at 1200 W is provided as supplemental data.

The microwave power required for a 5‐min DRX synthesis was re‐optimized for the Milestone reactor cavity. Figure S1 presents the sXRD patterns of samples heated for 5 min at microwave powers ranging from 600 to 1800 W. At low power (e.g., 600 W), impurity phases associated with incomplete DRX formation are observed (discussed later). Conversely, excessively high power (e.g., 1800 W) drives the formation of ordered Li–Mn–O phases. Intermediate microwave powers yield DRX with high phase purity; thus, 1200 W was chosen as the optimal power for subsequent reaction pathway analysis.

The 5‐min heating profile measured at the center of the pellet is shown in Figure 2a. Temperature recording began after 1 min after the onset of MW irradiation, once the pellet temperature surpassed the minimum detectable limit of 525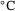. Two prominent features are observed in the heating profile, where the heating rate increases suddenly and dramatically: the first at approximately 700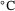 and the second at approximately 945 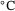.

**FIGURE 2:**
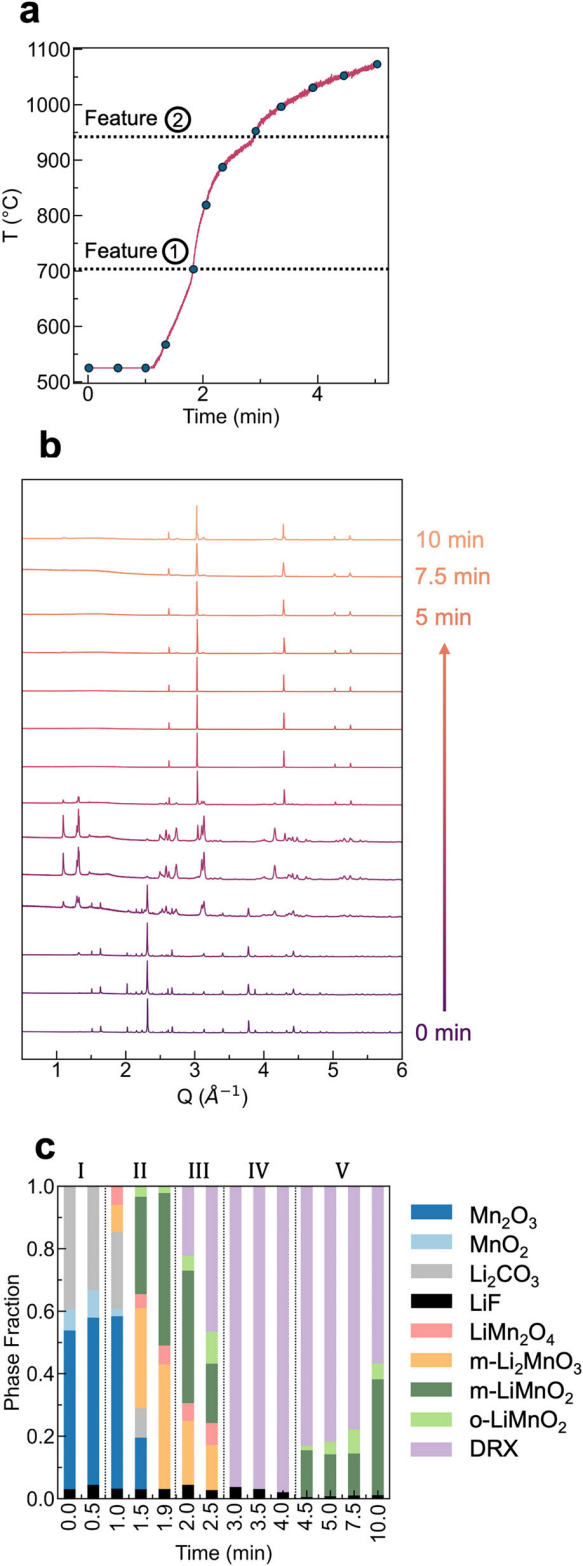
Ex situ reaction pathway analysis of LM9 precursor pellets heated at 1200 W. (a) IR temperature‐time profile recorded during microwave heating. Blue circles indicate the time points at which samples were quenched for *ex situ* analysis. (b) Corresponding synchrotron XRD patterns. (c) Phase mass fractions determined from multiphase Rietveld refinements. Dashed lines in (a) and (c) highlight the positions of key features in the IR profile and the corresponding stages of the reaction pathway, respectively.

The reaction pathway was investigated ex situ by quenching samples heated for various durations between 0 and 5 min at approximately 30 s intervals, with an additional sample collected at 1.9 min to coincide with the 700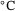 feature (Figure 2a). Two further samples were heated for extended durations of 7.5 and 10 min; the corresponding 10‐min heating profile is provided in the Supporting Information (Figure S2).

Aliquots of the intimately mixed precursor powders prepared by planetary mixing (t=0 min) and well‐ground quenched samples (0.5<t<10 min) were loaded into capillaries for sXRD measurements (Figure 2b). Multiphase Rietveld refinements were performed to identify and estimate the relative amounts of crystalline phases present in each sample, and their evolution was analyzed as a function of reaction time (Figure 2c). Fits, refinement parameters, and peak deconvolutions are provided in the Supporting Information (Figure S3). Although phase identification and relative trends can be reliably tracked, significant peak overlap limits the precision with which individual phase fractions can be determined. Despite careful efforts to minimize correlations between scale factors and other refinable parameters, extracted phase fractions should be regarded as semi‐quantitative indicators of phase evolution rather than absolute values, with emphasis placed on observed trends [45].

We find that the reaction pathway can be divided into the following five regions:

*Region I — Unreacted precursors*. The sharp Bragg reflections are attributed to the highly crystalline starting materials: ${\mathrm{Mn}}_{2}O_{3}$ (Pbca), ${\mathrm{MnO}}_{2}$ ($P4_{2}/mnm$), ${\mathrm{Li}}_{2}{\mathrm{CO}}_{3}$ (C2/m), and LiF ($Fm\bar{3}m$). Rietveld refinement against the t=0 data yields phase fractions in close agreement with the nominal weighed mass ratios of the reagents. The broad diffuse feature centered at 1.6 Å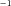 arises from background scattering of the amorphous borosilicate capillaries. After 0.5 min of heating, no measurable phase evolution is observed and the reaction temperature remains below the minimum detection threshold (T < 525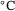).

*Region II — Formation of ordered ternary Li–Mn–O phases*. By 1.0 min (T ∼ 525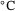), broad, low‐intensity reflections emerge that can be indexed to layered ${\mathrm{Li}}_{2}\mathrm{Mn}O_{3}$ and spinel ${\mathrm{LiMn}}_{2}O_{4}$ (8.7 and 6.0 wt%, respectively). At 1.5 min (T ∼ 600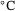), additional reflections corresponding to the two polymorphs of ${\mathrm{LiMnO}}_{2}$ (monoclinic m–${\mathrm{LiMnO}}_{2}$, and orthorhombic o−${\mathrm{LiMnO}}_{2}$) become apparent. Between 1.5 and 2.5 min (600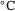 <T< 900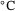), the background intensity increases markedly, which we attribute to diffuse scattering from poorly crystalline transient phases, increasing structural disorder, and/or ongoing microstructural evolution. By 1.9 min (T ∼ 760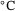), all starting reagents are consumed, and m–${\mathrm{LiMnO}}_{2}$ and ${\mathrm{Li}}_{2}\mathrm{Mn}O_{3}$ become the dominant phases (48.8 and 39.9 wt%, respectively), accompanied by minor fractions of o−${\mathrm{LiMnO}}_{2}$ and ${\mathrm{LiMn}}_{2}O_{4}$ spinel.

To complement sXRD, we performed 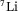 solid‐state NMR spectroscopy to obtain local structural information and to strengthen confidence in the intermediate phases incorporated into our multiphase Rietveld refinements. 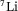 MAS NMR spectra of the intermediate samples (1.0–3.5 min) are shown in Figure S4. As a local probe sensitive to the immediate coordination environments of 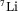, NMR spectroscopy is particularly valuable for unambiguous phase identification in poorly crystalline samples. Short recycle delays (20 ms) were used to suppress signals from diamagnetic Li species, aiding identification of Mn‐containing phases. This was especially important for the 1.0 min sample, in which the dominant Li‐containing phase remained the diamagnetic ${\mathrm{Li}}_{2}{\mathrm{CO}}_{3}$ precursor (for comparison, a 1.0 min spectrum collected with a 10 s recycle delay is shown in Figure S5).

The NMR spectra collected on samples stopped between 1.0 and 3.5 min reveal the concurrent emergence of multiple ternary intermediates that persist until DRX phase purity is achieved at 3.5 min. Peak assignments were established by fitting the 1.5 min spectrum (Figure S6) and corroborating the resulting resonances with prior literature reports. We note that the chemical shifts of the different environments depend on sample temperature and are measured here at a higher temperature (∼320 K) under 60 kHz magic‐angle spinning (MAS) than in earlier NMR studies of Li–Mn–O phases, which were typically performed at lower MAS frequencies (∼300 K). The resonance at −9 ppm is attributed to diamagnetic Li species (including ${\mathrm{Li}}_{2}{\mathrm{CO}}_{3}$ and LiF) [46, 47]; the resonance at 25 ppm to o−${\mathrm{LiMnO}}_{2}$; and the resonance at 117 ppm to m–${\mathrm{LiMnO}}_{2}$ [47, 48, 49, 50]. An intense resonance at 71 ppm indicates an intergrowth of these two polymorphs (Figure S4), by analogy with prior work [51] on the Na analogue, ${\mathrm{NaMnO}}_{2}$. In that system, the 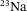 paramagnetic (Fermi contact) shift was decomposed into additive contributions from neighboring Mn centers via Mn–O–Na interaction pathways using bond pathway analysis [52] and first‐principles calculations. This analysis showed that Na environments in m−${\mathrm{NaMnO}}_{2}$ and o−${\mathrm{NaMnO}}_{2}$ differ in the number of 180‐degree Mn–O–Na bond pathways, with Na in m−${\mathrm{NaMnO}}_{2}$ environments exhibiting two additional 180‐degree pathways. These additional contributions account for the large shift separation at 4.7 T (751 ppm for m−${\mathrm{NaMnO}}_{2}$ vs. 318 ppm for o−${\mathrm{NaMnO}}_{2}$). A resonance at 528 ppm, intermediate between the two polymorphs, was observed for samples containing both structures and was assigned to Na near stacking faults characteristic of layered intergrowths, reflecting one additional 180‐degree bond pathway relative to Na in o−${\mathrm{NaMnO}}_{2}$ domains [51]. By direct analogy, the 71 ppm resonance observed here lies between the o−${\mathrm{LiMnO}}_{2}$ (25 ppm) and m–${\mathrm{LiMnO}}_{2}$ (117 ppm) resonances and is therefore attributed to Li near stacking faults.

Given the short synthesis times, m–${\mathrm{LiMnO}}_{2}$ forms with limited long‐range order, instead occurring as an intergrowth with o−${\mathrm{LiMnO}}_{2}$ related by a b/2 translation. The intergrowth perturbs the stacking sequence along the a axis, giving rise to broadened, asymmetric Bragg reflections with strong a‐axis contributions in the sXRD data, as reported by Reimers et al. [53] and subsequently modeled by Croguennec et al. [54]. The resulting structural disorder is reflected in the reduced goodness‐of‐fit values obtained from Rietveld refinements of the 1.9 and 2.0 min datasets. Such faulting has been associated with short reaction times and is often accompanied by the formation of ${\mathrm{LiMn}}_{2}O_{4}$ spinel [54].

Low‐intensity resonances between 300 and 600 ppm are assigned to spinel‐type phases. The resonance at 490 ppm corresponds to ${\mathrm{LiMn}}_{2}O_{4}$ spinel [46, 55], while the feature at 356 ppm is tentatively attributed to a distorted, partially disordered spinel‐like phase reported to coexist with ${\mathrm{LiMnO}}_{2}$ [49]. The asymmetric resonances near 700 and 1430 ppm are assigned to Li in the Li layers (4h and 2c sites) and Li in the Mn layers (2b sites) of ${\mathrm{Li}}_{2}\mathrm{Mn}O_{3}$, respectively [46, 56, 57]. Their pronounced asymmetry is indicative of faulting [57]. Overall, NMR confirms the phases used in the multiphase Rietveld refinements, and reveals defect environments in spinel‐like phases and ${\mathrm{LiMnO}}_{2}$ not captured by diffraction alone.

*Region III — DRX phase formation*. By 2.0 min, a crystalline DRX phase has emerged, and by 2.5 min, it has grown to become the dominant phase (800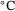 <T< 900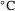).

*Region IV — Phase‐pure DRX*. Powder sXRD patterns collected on samples quenched at 3.0, 3.5, and 4.0 min (960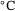 <T< 1040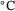) exhibit sharp, high‐intensity reflections, indicative of a high degree of crystallinity. The Bragg peaks are indexed primarily to the DRX phase, which constitutes 96.3–97.9 wt %, with a small amount of LiF identified by the weak but sharp reflections at 2.7 and 3.1 Å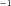 [42, 43].

Quantifying fluorine (F) incorporation in DRX materials is challenging as F cannot easily be distinguished from O by neither x‐ray nor neutron diffraction, and requires the use of complementary techniques including F‐ion selective electrode and inductively coupled plasma, 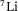 and 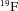 NMR measurements [58]. In a previous study, we showed that overall F incorporation decreases with increasing Mn content [44]. For LM9 specifically, our previous report demonstrated that approximately half of the F volatilizes during the synthesis, while only approximately one‐quarter to one‐half of the F in the precursor mixture is incorporated into the DRX structure; the remainder persists as a surface impurity phase [42]. Because LM9 was prepared using the same precursor mixture and synthesis procedure as in our earlier report, its F content was not quantified again in the present study.

Given the limited F incorporation in LM9, LiF is considered primarily for its role as a sintering agent that promotes the formation of a phase‐pure DRX, rather than as a meaningful contributor to the bulk composition [43]. Although a small degree of F incorporation may slightly lower the disordering temperature, as predicted from first principles for ${\mathrm{LiMnO}}_{2}$ containing $F_{\text{0.05}}$ [59] and as expected from the increased configurational entropy of the anion sublattice, the primary driver of disordering remains sintering at temperatures above the disordering transition. The exact disordering temperature depends on the composition and can vary with factors such as the extent of Li overstoichiometry and the concentration of $d^{0}$ transition metal cations [60]. We note that fully F‐free LM9 can be synthesized via microwave heating in the absence of LiF. However, under these conditions, small amounts of ordered impurity phases remain present (Figure S7).

*Region V — Re‐emergence of ordered Li–Mn–O phases*. For reaction times exceeding 4.5 min (T > 1050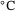), additional reflections appear at 1.1, 2.7, 3.1, and 4.2 Å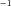, which are assigned to m–${\mathrm{LiMnO}}_{2}$ and o−${\mathrm{LiMnO}}_{2}$. Between 4.5 and 10 min, the relative fraction of the DRX phase decreases while the fractions of m–${\mathrm{LiMnO}}_{2}$ and o−${\mathrm{LiMnO}}_{2}$ increase, consistent with thermodynamically driven Li/Mn reordering on the cation sublattice. The proportion of m–${\mathrm{LiMnO}}_{2}$ remains consistently higher than that of o−${\mathrm{LiMnO}}_{2}$, reflecting the greater stability of the monoclinic phase, which more effectively accommodates Jahn–Teller distortions associated with ${\mathrm{Mn}}^{3+}$, as previously reported by Ceder and coworkers [61, 62]. As observed in region III, the reentrant layered ${\mathrm{LiMnO}}_{2}$ exhibits evidence of intergrowth between two polymorphs, manifested by asymmetric broadening of characteristic Bragg reflections in the sXRD data and the re‐emergence of the 71 ppm resonance in the 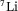 NMR spectrum (Figure 3). Furthermore, a weak feature near 700 ppm suggests the presence of a small amount of ${\mathrm{Li}}_{2}\mathrm{Mn}O_{3}$, likely below the detection limit of sXRD.

**FIGURE 3:**
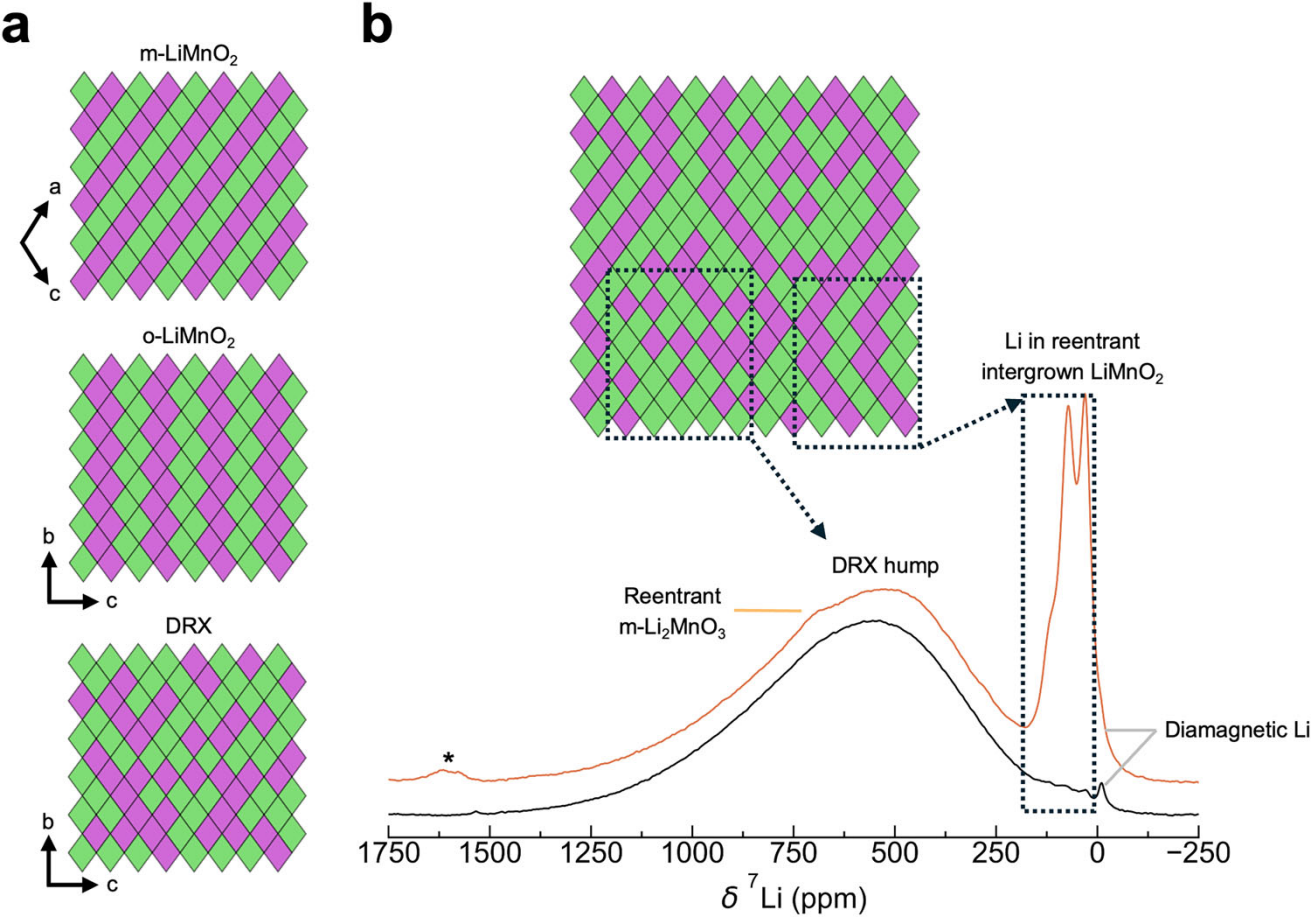
(a) Structural comparison of ordered ${\mathrm{LiMnO}}_{2}$ polymorphs and LM9 DRX. Disordering or reordering of the cations along the c‐axis enables interconversion between these structures. (b) 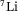 NMR spectra of LM9 heated for 3.5 min (black) and 10 min (orange), showing the re‐emergence of characteristic resonances assigned to Li in o−${\mathrm{LiMnO}}_{2}$ (25 ppm) and m−${\mathrm{LiMnO}}_{2}$ (117 ppm) domains, as well as Li within intergrowth regions (71 ppm). The structure may consist of a mosaic of coherent DRX and layered domains.

By 10 min, low intensity flat‐topped shoulders appear on the high‐Q side of the reflections associated with the DRX phase (Figure S8). These peak profiles suggest the superposition of two or more scattering contributions with similar d‐spacings. This result may stem from either (i) the nucleation of pseudo‐ordered nano‐domains (possibly cation ordering over an intermediate length scale), or (ii) a distribution of domains with slightly different lattice parameters or strain states.

To gain additional insight into the reentrant phase behavior, we examined the evolution of the average Mn oxidation state and Li content in samples quenched at 3.5 min of microwave heating and beyond. Mn K‐edge X‐ray absorption spectroscopy (XAS) results (Figure S9) show a progressive decrease in Mn oxidation state with extended heating time. Linear interpolation of the ${\mathrm{Mn}}^{3+/4+}$ edge positions relative to binary Mn–O reference samples indicates an average oxidation state of ${\mathrm{Mn}}^{3.16+}$ at 3.5 min (close to the ${\mathrm{Mn}}^{3.11+}$ value expected from the stoichiometric ratio of ${\mathrm{Mn}}^{3+/4+}$ precursors), which decreases to ${\mathrm{Mn}}^{2.98+}$ by 10 min. Meanwhile, elemental analysis reveals Li loss upon extended heating: the Li:Mn ratio is 1.20 for the 3.5 min sample (close to the target stoichiometric value of 1.22), but decreases to 1.13 after 10 min. One possible explanation for the observed Mn reduction and Li loss is the concurrent evolution of Li and oxygen from the lattice during overheating, potentially through the formation and volatilization of ${\mathrm{Li}}_{2}O$ or LiOH. Alternatively, these species may be melting into the ${\mathrm{Al}}_{2}O_{3}$ crucibles. This mechanism is discussed in greater detail in the Discussion section.

Finally, scanning electron microscopy (SEM) was performed on the full series of ex situ samples to track the evolution of particle size and morphology (Figure S10). From 0 to 1.0 min of heating, the precursor particles remain large and jagged, consistent with the relatively sharp and unchanged sXRD reflections. The first major morphological change occurs at 1.5 min, when thin, nanoscale lamellae appear on the surface of the particles. These features likely arise from the formation of layered intermediates (${\mathrm{Li}}_{2}\mathrm{Mn}O_{3}$, m−${\mathrm{LiMnO}}_{2}$, and o−${\mathrm{LiMnO}}_{2}$), which also give rise to the broadened reflections observed in the diffraction profiles. With further heating (1.9–2.5 min), the lamellae grow and coalesce into agglomerates of larger, plate‐like primary particles, before transforming into the target DRX phase by 3.0 min. Prolonged heating leads to continued DRX grain growth and particle coarsening (Figure S11). Analyses of SEM micrographs collected on samples that had been microwave irradiated for 3.5 and 7.5 min, hereafter referred to as LM9‐3.5 and LM9‐7.5, respectively, yield median particle sizes (d50) of 1.03 and 2.40 μm, respectively. Additional particle size metrics are summarized in Table 1.

**TABLE 1:** Particle size distributions for LM9‐3.5 and LM9‐7.5.

Sample	Average (μm)	Std. Dev. (μm)	d10 (μm)	d50 (μm)	d90 (μm)	Span
LM9‐3.5	1.189	0.696	0.503	1.029	2.059	1.512
LM9‐7.5	2.741	1.787	1.212	2.396	4.587	1.409

### 2.2 Chemical Origin of Infrared Profile Features

IR thermal profiles for DRX syntheses performed using a microwave power level between 600 and 1800 W are shown in Figure 4a. As mentioned earlier, the thermal profiles exhibit two prominent features, referred to as feature 1 and 2, hereafter. By examining the power dependence of each feature, their chemical origin can be elucidated.

**FIGURE 4:**
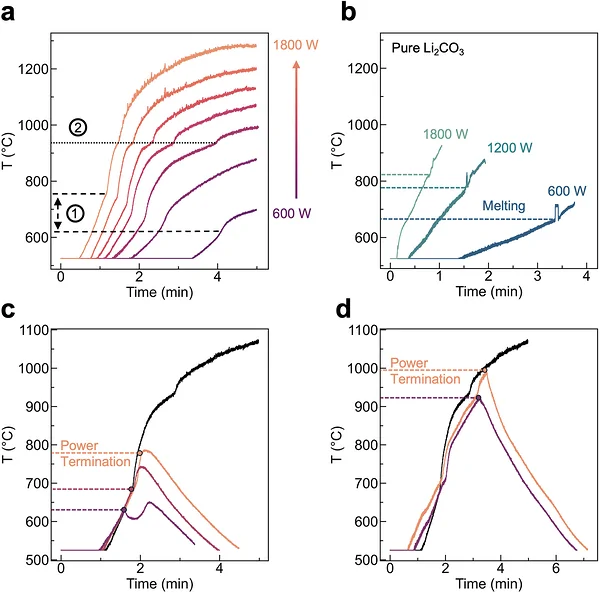
(a) IR temperature‐time profiles of LM9 precursor pellets heated under microwave powers ranging from 600 to 1800 W, followed by quenching in water after 5 min. Features 1 and 2 are indicated by dashed and dotted lines, respectively. (b) IR temperature‐time profiles of pure ${\mathrm{Li}}_{2}{\mathrm{CO}}_{3}$ pellets under varying microwave powers. The melting point of ${\mathrm{Li}}_{2}{\mathrm{CO}}_{3}$ is marked by a corresponding dashed line. (c,d) IR temperature‐time profiles of LM9 precursor pellets heated at 1200 W, in which microwave power was intentionally terminated (denoted by dashed lines and circles) and the subsequent temperature evolution was monitored in situ without quenching. Panel (c) corresponds to power termination near feature 1, and panel (d) to termination near feature 2.

Feature 1 appears strongly power dependent, shifting by as much as 135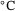 with applied microwave power (from 625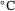 at 600 W to 760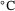 at 1800 W), whereas feature 2 consistently occurs at 945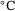, independent of power. The power‐dependence of feature 1 suggests a kinetically limited process resembling a second‐order phase transition (e.g., melting). Based on the evolution of phase mass fractions upon heating at 1200 W (Figure 2c), this feature seems to coincide with melting of the ${\mathrm{Li}}_{2}{\mathrm{CO}}_{3}$ precursor (nominal melting temperature $T_{m}$ $∼700^{\circ }C$). To test whether melting of ${\mathrm{Li}}_{2}{\mathrm{CO}}_{3}$ underlies this power‐dependent behavior, pure ${\mathrm{Li}}_{2}{\mathrm{CO}}_{3}$ pellets were heated at 600, 1200, and 1800 W (Figure 4b). IR thermal imaging shows that the ${\mathrm{Li}}_{2}{\mathrm{CO}}_{3}$ pellets melt with a similar power dependence and over a comparable temperature range to feature 1 observed for LM9 pellets. Hence, we attribute feature 1 to melting of ${\mathrm{Li}}_{2}{\mathrm{CO}}_{3}$ during synthesis. We note that the IR temperature calibration assumes an emissivity of 1 (corresponding to an ideal black body). While this assumption is reasonably accurate for the black DRX pellets, it leads to a systematic overestimation of the temperature of the white ${\mathrm{Li}}_{2}{\mathrm{CO}}_{3}$ pellets.

We next examined the thermodynamics of ${\mathrm{Li}}_{2}{\mathrm{CO}}_{3}$ melting and how they are reflected in the IR thermal profiles. In doing so, we evaluated additional factors that could affect the measured temperature evolution during synthesis, including direct microwave coupling to the alumina crucible or the DRX pellet, and potential temperature‐dependent changes in emissivity. We determined that none of these effects significantly affect the observed thermal features. Details are provided in Note S2 and Figures S12 and S13.

Phase transitions from solid to liquid consume heat; accordingly, the latent heat of melting of ${\mathrm{Li}}_{2}{\mathrm{CO}}_{3}$ should appear as a distinct feature in the thermal profile. Once molten, ${\mathrm{Li}}_{2}{\mathrm{CO}}_{3}$ is expected to exhibit a significantly higher chemical potential, which would promote rapid reaction with manganese oxide precursors and facilitate the formation of ternary Li–Mn–O phases. To isolate this thermic event from the continuous input from microwave heating, the surface temperature of a series of DRX precursor pellets was monitored during irradiation at 1200 W. In each case, the microwave power was terminated just above, at, or just below feature 1 (observed at 680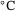 under this power condition). Any spontaneous exothermic or endothermic process occurring after power cutoff would be reflected in the resulting temperature profile (Figure 4c). When the power was terminated above feature 1, the pellet exhibited simple radiative cooling to room temperature, with no evidence of additional thermic events. In contrast, terminating the power precisely at feature 1 (680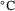) led to the spontaneous generation of heat, increasing the pellet surface temperature by an additional $∼60^{\circ }C$ before it cooled to room temperature. When the power was cut just below the transition, the pellet temperature first dropped sharply by $∼25^{\circ }C$, consistent with heat absorption, before plateauing and subsequently increasing by $∼45^{\circ }C$. After this secondary rise, the pellet cooled monotonically to room temperature. These observations indicate that immediately below feature 1, the rate of temperature increase is suppressed due to endothermic melting of ${\mathrm{Li}}_{2}{\mathrm{CO}}_{3}$. Once melting is complete, the molten ${\mathrm{Li}}_{2}{\mathrm{CO}}_{3}$ possesses a substantially higher chemical potential, which promotes exothermic reactions with the manganese oxide precursors. This reaction‐driven heat release explains the rapid temperature increase above feature 1.

The precise origin of feature 2 at 945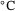 is less obvious. By this stage of the reaction, the DRX phase constitutes the majority phase, leading us to hypothesize that this feature corresponds to energy absorption associated with disordering of the remaining intermediate phases to form the DRX. This is difficult to demonstrate experimentally. As shown in Figure 4d, any latent heat associated with this transformation is insufficient to be resolved by terminating microwave power just below or above feature 2 and monitoring the subsequent surface temperature evolution of the pellet. Unlike feature 1, no clear exo‐ or endothermic event is detectable through this approach. We therefore turn to XRD for further insight. The evolution of phase mass fractions during heating (Figure 2c) indicates that feature 2 marks the transition from a multiphase mixture (DRX and ordered intermediates) to a single‐phase DRX material. Quenched samples collected immediately below and above this feature confirm that it consistently indicates complete DRX formation at all microwave power levels capable of heating the pellet to 945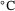 (Figure 5). Accordingly, feature 2 serves as a reliable in situ indicator of reaction completion during DRX microwave synthesis.

**FIGURE 5:**
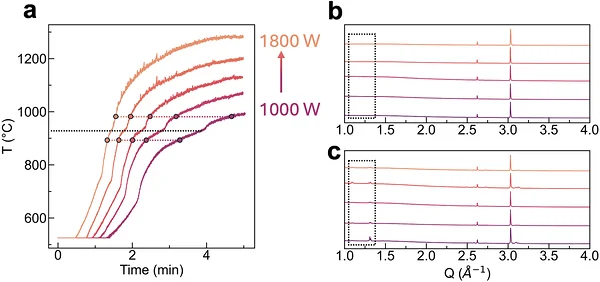
(a) IR temperature‐time profiles of LM9 precursor pellets heated under microwave powers ranging from 1000 to 1800 W. The circles indicate the temperatures at which pellets were quenched for ex situ phase analysis, either just above (980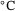) or just below (900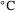) feature 2 at 945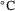. Synchrotron XRD patterns of samples quenched at (b) 980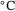 and (c) 900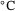 are shown. The dotted boxes in (b,c) highlight the region containing impurity reflections, which are observed in the 900
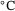
‐quenched samples but are absent in those quenched at 980 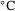. Full XRD patterns are provided in Figure S14.

### 2.3 Impact of Cation Reordering on Electrochemical Performance

Mn‐rich DRX undergo a structural transformation during electrochemical cycling, characterized by local cation rearrangements that produce nanodomains with spinel‐like cation ordering separated by antiphase boundaries [63]. The resulting structure is commonly referred to as the “δ‐phase” [63, 64, 65, 66]. This transformation is accompanied by significant capacity activation, attributed to enhanced Li transport within the spinel domains [67]. Because the δ‐phase transformation proceeds sluggishly under conventional galvanostatic cycling, several strategies have been developed to accelerate its formation. These include chemical delithation followed by annealing [63, 65], and electrochemical pulse conditioning at elevated temperature [68], the latter of which is employed in the present work.

Two samples were selected for electrochemical evaluation: LM9‐3.5, corresponding to the maximum DRX phase purity (97 wt%; median particle size ∼1.0 μm), and LM9‐7.5, containing 78 wt% DRX phase and 22 wt% ${\mathrm{LiMnO}}_{2}$, with a median particle size of ∼2.4 μm). Electrochemical testing was conducted using half‐cells subjected to an electrochemical pulse formation protocol (50 pulses between 3.5 and 4.5 V at C/2, with the cell held at 50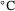 in a temperature‐controlled chamber; a procedure adapted from Wu et al. [42].). Following formation, the cells were cycled at a current density of 20 mA/g (approximately C/10) over a voltage window of 1.5–4.8 V. The corresponding voltage profiles for LM9‐3.5 and LM9‐7.5 are presented in Figure 6a and Figure 6b, respectively.

**FIGURE 6:**
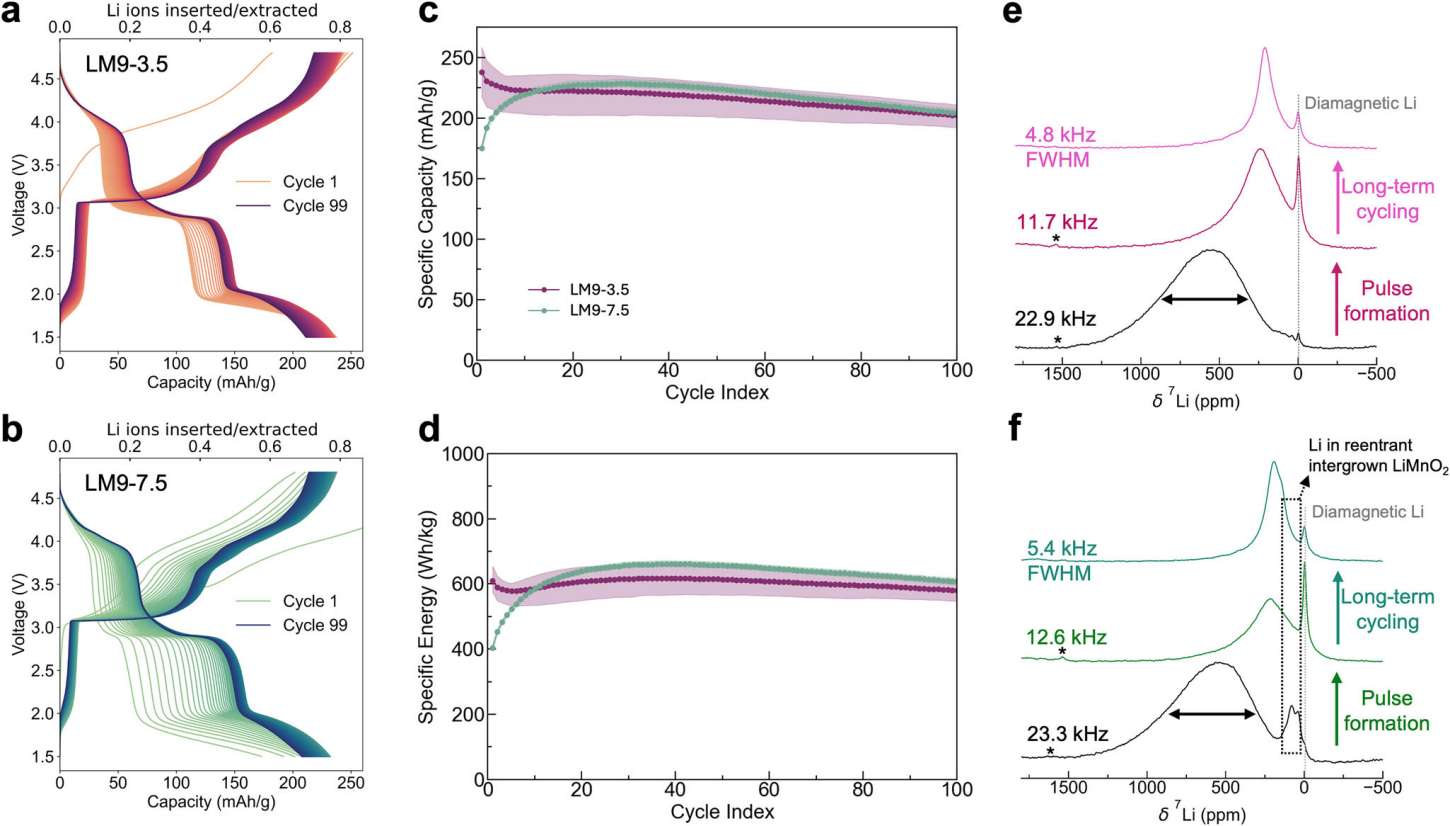
Voltage profiles of Li‐ion half cells comprising an LM9 cathode synthesized using a (a) 3.5 and (b) 7.5 min microwave heating time (LM9‐3.5 and LM9‐7.5, respectively) at 10 mA/g current density. (c) Average specific capacity and (d) average specific energy as a function of cycle number. The circular markers represent the average of three cells for each LM9 cathode, and shaded envelopes represent the maximum and minimum capacities achieved by any one of the triplicate cells. (e,f) Ex situ 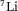 NMR of cathode films recovered at various stages of cycling (Pristine, post pulse‐formation, and post 100 cycles) for LM9‐3.5 and LM9‐7.5, respectively. The full width at half maximum (FWHM) of the broad DRX feature is annotated to the left of each spectrum.

Both materials undergo only partial δ‐phase transformation during pulse formation, with LM9‐3.5 exhibiting a greater extent of transformation. This difference is reflected in the voltage profiles: the LM9‐3.5 profile remains largely stable upon cycling, aside from a modest extension of the ∼4 V pseudo‐plateau, consistent with further cation reordering toward a spinel‐like configuration. In contrast, the LM9‐7.5 voltage profile evolves dramatically over the first 30 cycles. The initial large capacity below 2 V (102 mAh $g^{-1}$ in the first cycle) decreases to 70 mAh $g^{-1}$ by cycle 30, accompanied by substantial growth of both the 4 V pseudo‐plateau and the 3 V plateau, each characteristic of spinel‐like cation ordering. After 30 cycles, however, the voltage profiles of LM9‐3.5 and LM9‐7.5 converge, as evidenced by their similar evolution from cycles 30–100 and their comparable dQ/dV features (Figure S15). Taken together, these results indicate that LM9‐7.5 undergoes a less complete δ‐phase transformation during pulse formation than LM9‐3.5.

The cycling performance as a function of cycle number was averaged over triplicate cells and is shown in Figure 6c,d. Although LM9‐7.5 exhibits lower initial capacity and energy, it undergoes pronounced activation, whereas LM9‐3.5 experiences a slight capacity loss during the early cycles before stabilizing by cycle 30. The average first‐cycle capacities of LM9‐3.5 and LM9‐7.5 are 238 and 175 mAh/g, respectively, evolving to 221 and 229 mAh/g by cycle 30. A noticeable spread in LM9‐3.5 cell performance is evident from the purple shaded envelope in Figure 6c,d. Notably, the top‐performing cells of LM9‐3.5 and LM9‐7.5 show nearly identical performance and cycling stability from cycle 30 onward, delivering 202 mAh/g and 203 mAh/g, respectively, at cycle 100. Relative to their highest‐capacity cycles (cycle 1 for LM9‐3.5 and cycle 30 for LM9‐7.5), capacity retention at cycle 100 is 85% and 89%, respectively.

The rate performance of LM9‐3.5 and LM9‐7.5 half‐cells was evaluated both immediately after formation and after 100 cycles (Figure S16). Initial rate testing reveals that LM9‐7.5 exhibits inferior rate capability compared to LM9‐3.5, likely due to an incomplete δ‐phase transformation during pulse formation. After 100 cycles, however, the rate performance more accurately reflects the intrinsic rate capabilities of the materials, as all cells have fully completed the structural transition. At low to intermediate current densities (20, 50, 100, 200 mA/g, approximately C/10, C/4, C/2, and 1C), the average capacities of LM9‐3.5 and LM9‐7.5 are similar. However, when comparing the best‐performing cells from each set, LM9‐7.5 shows superior rate capability at high current densities (400 and 1000 mA/g, approximately 2C and 5C).

The electrochemical results indicate that delayed capacity activation, arising from delayed δ‐phase transformation, is the primary consequence of overheating beyond the point of maximum DRX phase purity. However, it remains unclear whether the increased particle size (Table 1), the re‐emergence of ordered ${\mathrm{LiMnO}}_{2}$, or a combination of both factors, is responsible for delaying the δ‐phase transformation.


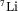
 NMR spectra collected on pristine and ex situ discharged LM9‐3.5 and LM9‐7.5 samples confirm partial conversion to the δ‐phase following pulse formation, as evidenced by the shift and sharpening of the broad DRX resonances (Figure 6e,f). Notably, resonances attributed to intergrown ${\mathrm{LiMnO}}_{2}$ disappear from the LM9‐7.5 spectrum, indicating that these domains also participate in the structural rearrangements. We exclude the possibility that these environments simply delithiate and become electrochemically inactive, as LM9‐7.5 retains a high reversible capacity comparable to phase‐pure LM9‐3.5. Furthermore, ${\mathrm{LiMnO}}_{2}$ is known to convert to ${\mathrm{LiMn}}_{2}O_{4}$ spinel during electrochemical cycling [69, 70]. Prolonged cycling further narrows the residual broad DRX resonance, consistent with continued structural reordering, a behavior previously observed in LM9 [42].

### 2.4 Discussion

The microwave reaction pathway of LM9 is analogous to that reported for the ${\mathrm{Li}}_{1.1}{\mathrm{Mn}}_{0.8}{\mathrm{Ti}}_{0.1}O_{1.9}F_{0.1}$ (LMT81) DRX phase by Liang et al. using in situ XRD during conventional furnace synthesis [71]. All ordered ternary Li–Mn–O intermediates identified during the microwave synthesis of LM9 were likewise observed in the furnace synthesis of LMT81, which additionally proceeds through a monoclinic ${\mathrm{Li}}_{2}$(MnTi)$O_{3}$ intermediate [71]. This similarity in reaction pathways is not surprising given the close compositional relationship between LMT81 and LM9, the principal difference being substitution of $d^{0}$ ${\mathrm{Ti}}^{4+}$ with $d^{3}$ ${\mathrm{Mn}}^{4+}$. However, reentrant phase behavior has not been reported for LMT81. This difference may suggest that the $d^{0}$‐free LM9 DRX framework is comparatively less stable, as reflected by its tendency to reorder into ${\mathrm{LiMnO}}_{2}$.

The order‐to‐disorder transition during LM9 synthesis is readily rationalized by prior computational work from Ceder and coworkers, who predicted disordering temperatures of approximately 1200–1350 K (∼930
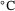
–1080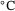) for ${\mathrm{Li}}_{1+x}{\mathrm{Mn}}_{1-x}O_{2-2x}F_{2x}$ compositions with 0≤x≤0.05 [59]. This temperature range encompasses the temperature at which LM9 (x = 0.05) phase purity is experimentally established (945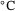).

In contrast, the reentrant formation of ${\mathrm{LiMnO}}_{2}$ upon overheating is less straightforward to rationalize. Reentrant phase transitions are relatively uncommon and typically involve the reemergence of a specific crystal structure, most often in systems governed by magnetic or optical ordering phenomena [72, 73, 74]. A closely related structural example is the reentrant order–disorder–order transition in ${\mathrm{MgAl}}_{2}O_{4}$ spinel, in which the degree of Mg/Al cation ordering and inversion on the spinel sublattice varies non‐monotonically with temperature [75]. In LM9, the analogous order–disorder–order transition corresponds to the rearrangement of Li and Mn cations into, or out of, planar (m−${\mathrm{LiMnO}}_{2}$) or corrugated (o−${\mathrm{LiMnO}}_{2}$) layers perpendicular to the c‐axis. Importantly, both ${\mathrm{LiMnO}}_{2}$ polymorphs are ordered superstructures of the rocksalt framework, such that the close‐packed oxygen sublattice remains intact during cation reorganization (Figure 3). This preservation of the anion framework mirrors the structural continuity observed in the reentrant transition of ${\mathrm{MgAl}}_{2}O_{4}$.

The reentrant formation of ${\mathrm{LiMnO}}_{2}$ occurs concurrently with Mn reduction and Li loss, as evidenced by Mn K‐edge XAS and ICP analyses of samples quenched after 3.5 and 10 min of microwave heating. As mentioned earlier, LM9‐3.5 is phase‐pure, whereas LM9‐10 contains a mixture of LM9 DRX and ${\mathrm{LiMnO}}_{2}$. To account for both Mn reduction and Li depletion, we propose the following reaction mechanism:

(1) $$\begin{array}{c} {\mathrm{Li}}_{1.1}{\mathrm{Mn}(\mathrm{III})}_{0.8}{\mathrm{Mn}(\mathrm{IV})}_{0.1}O_{1.9}F_{0.1}⟶\\ 0.9{\mathrm{LiMn}(\mathrm{III})O}_{2}+0.1\mathrm{LiF}+0.05\mathrm{Li}2O+0.025O_{2} \end{array}$$

${\mathrm{Li}}_{2}O$ is expected to volatilize under microwave heating conditions. Computational simulations by Ceder and coworkers indicate that decreasing the Li and F content in ${\mathrm{Li}}_{1+x}{\mathrm{Mn}}_{1-x}O_{2-2x}F_{2x}$ raises the disordering temperature [59]. Hence, progressive Li and O loss during extended microwave irradiation is expected to shift the thermodynamic balance toward the ordered state as the pellet temperature stabilizes (Figure S2). The reentrant behavior is therefore likely thermodynamically driven, arising from a gradual compositional drift of the DRX phase during heating that reverses the order–disorder preference and promotes the formation of layered ${\mathrm{LiMnO}}_{2}$, particularly near particle surfaces where Li and O loss is most pronounced.

The IR thermal profiles recorded on DRX pellets during microwave heating provide additional insight into the synthesis process, successfully capturing the initial order‐to‐disorder transition that establishes a phase‐pure DRX. The in situ thermal signature of DRX phase purity offers a powerful and rapid screening tool for candidate compositions, particularly as the accessible chemical space has expanded with the recent identification of $d^{0}$‐free DRX [42]. A similar signature correlated with DRX phase purity was recently reported by Park et al. during the Joule‐heating synthesis of the ${\mathrm{Li}}_{1.2}{\mathrm{Mn}}_{0.4}{\mathrm{Ti}}_{0.4}O_{2}$ DRX [39]. However, in that study as well as in earlier reports employing IR pyrometry during microwave synthesis of other inorganic materials [7], the origins of the observed thermal features were not systematically deconvoluted and were largely attributed to spontaneous thermal events. Our results provide experimental validation that these features indeed arise from thermic events, while further distinguishing whether they are kinetically limited (e.g., the heating power dependence associated with ${\mathrm{Li}}_{2}{\mathrm{CO}}_{3}$ melting and decomposition) or thermodynamically limited (e.g., the power‐independent feature marking DRX phase purity). Together, these findings underscore the broad utility of IR pyrometry for mechanistic insight into solid‐state synthesis, particularly in rapid or nonequilibrium synthesis routes such as microwave or Joule‐heating, when conventional in situ characterization methods developed for furnace heating are less readily applicable.

In situ IR thermal profiles provide a measure of control over synthesis and the resulting DRX phase purity by enabling precise regulation of thermal exposure following phase formation. The importance of synthetic control over particle size, uniformity, and distribution has been emphasized in recent work [76]. In conventional furnace‐based synthesis of $d^{0}$‐containing DRX compositions, particle size can be tuned through calcination time and temperature, with reported sizes below 200 nm up to 1 μm [77]. In the present microwave synthesis, particle size is instead governed by the duration of heating after the onset of phase‐pure DRX formation (at $∼945^{\circ }C$). Since in situ IR monitoring identifies this transition in real time, subsequent thermal exposure can be precisely timed. Heating beyond this point primarily promotes particle coarsening and, if extended, ultimately leads to loss of DRX phase purity. Within this constrained window, increasing the heating time at 1200 W from 3.5 to 7.5 min results in an approximate doubling of the median particle size, from ∼1.0 to 2.4 μm (Figure S11). These findings demonstrate that in situ IR monitoring enables modulation of particle size through controlled post‐formation thermal exposure, while preserving the dominant DRX phase and avoiding decomposition or reentrant transformation.

Although extended heating reduces phase purity through the reentrant formation of layered ${\mathrm{LiMnO}}_{2}$ phases, subsequent electrochemical testing demonstrates that overall performance remains largely resilient to these structural changes. This robustness stems from the propensity of ternary Li–Mn–O phases, including both ${\mathrm{LiMnO}}_{2}$ and Mn‐rich DRX, to undergo an electrochemically driven transformation into the ${\mathrm{LiMn}}_{2}O_{4}$ spinel or a spinel‐like δ‐phase. As a result, despite a slower transition to the δ‐phase, the electrochemical performance of LM9‐7.5, which contains some amount of layered ${\mathrm{LiMnO}}_{2}$, reaches that of the phase‐pure LM9‐3.5 DRX by cycle 30. Furthermore, ex‐situ analysis of electrochemically transformed LM9‐3.5 and LM9‐7.5 samples indicates that the cathode structures become similar by cycle 30.

While ${\mathrm{LiMnO}}_{2}$ is known to transform into ${\mathrm{LiMn}}_{2}O_{4}$ spinel [69], the resulting spinel phase has a high density of defects and antiphase boundaries [78, 79]. Similarly, Mn‐rich $d^{0}$‐containing DRX (e.g., LMT81) undergo a δ‐phase transformation whereby spinel nanodomains nucleate throughout the bulk particle. Li and Mn migration drives continued growth of these nanodomains, which eventually impinge upon one another, giving rise to antiphase boundaries that are pinned by the less mobile $d^{0}$ ions [63, 66]. Notably, a recent study correlated the presence of antiphase boundaries in a δ‐transformed DRX (with a target composition of ${\mathrm{Li}}_{1.2}{\mathrm{Mn}}_{0.65}{\mathrm{Ti}}_{0.15}O_{1.9}F_{0.1}$ and spinel nanodomains of ∼3–7 Å in size) with suppression of the two‐phase (de)lithiation reaction at ∼3 V characteristic of conventional ${\mathrm{LiMn}}_{2}O_{4}$ spinel, a process commonly linked to electrochemical degradation [63].

In $d^{0}$‐free LM9, the growth of spinel domains likely proceeds through nucleation and growth, giving rise to a similar nanodomain structure with antiphase boundaries separating adjacent domains. However, in the absence of $d^{0}$ cation pinning, the mobility of these boundaries (and the resulting implications for structural stability and ${\mathrm{Li}}^{+}$ transport within the δ‐phase) remain unclear. Notably, a higher fraction of layered impurities in the as‐synthesized LM9 sample produces only minor changes in the shape of the voltage profile following the δ‐transition. This observation is consistent with the ex situ 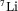 NMR results, which indicate the disappearance of the intergrown ${\mathrm{LiMnO}}_{2}$ resonances upon cycling. Orthorhombic and monoclinic layered ${\mathrm{LiMnO}}_{2}$ cathodes are known to undergo a phase transformation to a ${\mathrm{Li}}_{x}{\mathrm{Mn}}_{2}O_{4}$‐like spinel similar to the δ‐transition in the Mn‐rich DRX [47, 70, 80]. Because these transformations ultimately produce structurally similar spinel‐like phases, it is reasonable to expect the voltage profiles of LM9‐3.5 and LM9‐7.5 to converge despite differences in phase purity and, consequently, in the initial distribution of Li and Mn amongst the octahedral cation sites within the rocksalt‐type structures. Indeed, the δ‐LM9 voltage profiles reported here are similar to those of ${\mathrm{LiMnO}}_{2}$ cathodes [47, 70, 80]. The presence of layered ${\mathrm{LiMnO}}_{2}$ impurities in‐LM9 appears to primarily impact the kinetics of the δ‐phase transformation, the resulting electrochemical rate performance and long‐term cycling stability, rather than the equilibrium electrochemical behavior of the transformed phase. We hypothesize that this effect arises from differences in the size of the spinel domains formed from layered regions relative to those originating from the DRX matrix. Future work will seek to clarify these points.

## 3 Conclusion

This work investigates the microwave synthesis pathway of the $d^{0}$‐free disordered rocksalt (DRX) ${\mathrm{Li}}_{1.1}{\mathrm{Mn}}_{0.9}O_{1.9}F_{0.1}$ (LM9). Using ex situ synchrotron x‐ray diffraction (sXRD) and 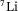 solid‐state NMR spectroscopy, we show that the reaction proceeds through five distinct stages: (i) initial heating below the reactivity threshold of the precursors, (ii) onset of reagent consumption to form ordered ternary Li–Mn–O intermediates, particularly ${\mathrm{LiMnO}}_{2}$, (iii) conversion of these intermediates into the DRX phase, (iv) a brief interval of high DRX phase purity, and (v) reentrant formation of ${\mathrm{LiMnO}}_{2}$ upon extended heating. In situ thermal profiles acquired via IR imaging reveal two thermic events during synthesis, corresponding to ${\mathrm{Li}}_{2}{\mathrm{CO}}_{3}$ melting and subsequent decomposition, and to the establishment of DRX phase purity, respectively. The identification of an in situ thermal signature marking phase‐pure DRX formation is especially valuable for rapid screening of new candidate DRX compositions within the rapidly expanding chemical space of $d^{0}$‐free systems. Electrochemical evaluation shows that, aside from delayed activation associated with sluggish transformation to the spinel‐like δ‐phase in LM9 samples containing reentrant layered ${\mathrm{LiMnO}}_{2}$ phases, overall performance converges with that of phase‐pure LM9 by cycle 30. These results indicate that the electrochemical performance of LM9 is relatively tolerant to variations in microwave synthesis conditions. Overall, this work presents the first reported case of reentrant phase formation during transition metal oxide synthesis, and establishes infrared pyrometry as a powerful tool for elucidating reaction pathways during solid‐state synthesis.

## Author Contributions

**Erick A. Lawrence**: conceptualization, methodology, formal analysis, investigation, visualization, writing – original draft, writing – review and editing. **Matthew A. Wright**: investigation, data analysis, writing – original draft, writing – review and editing. **Tianyu Li**: investigation, data analysis, writing – review and editing. **Euan N. Bassey**: investigation, data analysis, writing – review and editing. **Vijay Kumar**: investigation, writing – review and editing **Shiyu Yuan**: investigation, data analysis, writing – review and editing. **Yangying Zhu**: supervision, writing – review and editing. **Pierre‐Etienne Cabelguen**: supervision, writing – review and editing. **Raphaële J.Clément**: conceptualization, funding acquisition, supervision, writing – review and editing

## Conflicts of Interest

The authors declare no conflicts of interest.

## Supporting information




**Supporting File 1**: anie73765‐sup‐0001‐SuppMat.pdf.

**Supporting File 2**: anie73765‐sup‐0002‐VideoS1.mp4.

## Data Availability

The data that support the findings of this study are available from the corresponding author upon reasonable request.

## References

[1] M. Bacci , M. Bini , A. Checcucci , et al., “Microwave Heating for the Rapid Determination of Thermodynamic Functions of Chemical Reactions,” Journal of the Chemical Society, Faraday Transactions I 77 (1981): 1503–1509.

[2] R. J. Giguere , T. L. Bray , S. M. Duncan , and G. Majetich , “Application of Commercial Microwave Ovens to Organic Synthesis,” Tetrahedron Letters 27 (1986): 4945–4948.

[3] C. O. Kappe , “Controlled Microwave Heating in Modern Organic Synthesis,” Angewandte Chemie ‐ International Edition 43 (2004): 6250–6284. 10.1002/anie.200400655 15558676

[4] H. J. Kitchen , S. R. Vallance , J. L. Kennedy , et al., “Modern Microwave Methods in Solid‐State Inorganic Materials Chemistry: From Fundamentals to Manufacturing,” Chemical Reviews 114 (2014): 1170–1206. 10.1021/cr4002353 24261861

[5] M. Baghbanzadeh , L. Carbone , P. D. Cozzoli , and C. O. Kappe , “Microwave‐Assisted Synthesis of Colloidal Inorganic Nanocrystals,” Angewandte Chemie ‐ International Edition 50 (2011): 11312–11359. 10.1002/anie.201101274 22058070

[6] M. P. Selvam and K. J. Rao , “Microwave Synthesis and Consolidation of Gadolinium Aluminum Perovskite, a Ceramic Extraordinaire,” Advanced Materials 12 (2000): 1621–1624.

[7] J. Jamboretz , Y. Zhu , R. James , L. Mu , and C. S. Birkel , “Microwave‐Assisted Synthesis of P2‐ and O3‐type ${\mathrm{Na}}_{x}{\mathrm{COO}}_{2}$ Cathode Materials Studied by In Situ Raman Spectroscopy,” Chemistry of Materials 36 (2024): 8447–8457.

[8] A. R. Reach , A. Zohar , M. A. Wright , T. Li , and R. Seshadri , “Rapid Microwave Preparation of $\mathrm{Al}{\mathrm{Nb}}_{11}O_{29}$ and ${\mathrm{Ti}}_{2}{\mathrm{Nb}}_{10}O_{29}$ Wadsley‐Roth Lithium‐Ion Anode Compounds From Parent Oxides,” ACS Applied Energy Materials 8 (2025): 7714–7721.

[9] A. R. Reach , M. A. Wright , A. S. Mulligan , et al., “Enhanced Sodium Dynamics in Biphasic Nasicon Solid Electrolytes,” Chemistry of Materials (2026).

[10] O. Palchik , R. Kerner , Z. Zhu , and A. Gedanken , “Preparation of ${\mathrm{Cu}}_{\left(2-x\right)}\mathrm{Te}$ and HgTe by Using Microwave Heating,” Journal of Solid State Chemistry 154 (2000): 530–534.

[11] R. Harpeness and A. Gedanken , “Microwave‐Assisted Synthesis of Nanosized ${\mathrm{Bi}}_{2}{\mathrm{Se}}_{3}$ ,” New Journal of Chemistry 27 (2003): 1191–1193.

[12] Y. Zhao , J. M. Hong , and J. J. Zhu , “Microwave‐Assisted Self‐Assembled ZnS Nanoballs,” Journal of Crystal Growth 270 (2004): 438–445.

[13] W. Yang , L. Zhang , Y. Hu , Y. Zhong , H. B. Wu , and X. W. Lou , “Microwave‐Assisted Synthesis of Porous ${\mathrm{Ag}}_{2}S$‐Ag Hybrid Nanotubes With High Visible‐Light Photocatalytic Activity,” Angewandte Chemie ‐ International Edition 51 (2012): 11501–11504. 10.1002/anie.201206715 23060047

[14] A. Agostino , P. Volpe , M. Castiglioni , and M. Truccato , “Microwave Synthesis of ${\mathrm{MgB}}_{2}$ Superconductor,” Materials Research Innovations 8 (2004): 75–77.

[15] T. Baldridge and M. C. Gupta , “Zirconium Diboride Nanofiber Generation via Microwave Arc Heating,” Nanotechnology 19 (2008): 275601. 10.1088/0957-4484/19/27/275601 21828708

[16] R. Ahlers and U. Ruschewitz , “Zur Synthese Von Binären und Ternären Carbiden in Einem Modifizierten Haushaltsmikrowellenofen,” Zeitschrift fur Anorganische und Allgemeine Chemie 631 (2005): 1241–1246.

[17] S. R. Vallance , D. M. Round , C. Ritter , E. J. Cussen , S. Kingman , and D. H. Gregory , “Ultrarapid Microwave Synthesis of Superconducting Refractory Carbides,” Advanced Materials 21 (2009): 4502–4504.

[18] B. Vaidhyanathan and K. J. Rao , “Microwave Assisted Synthesis of Technologically Important Transition Metal Silicides,” Journal of Materials Research 12 (1997): 3225–3229.

[19] J. R. Jokisaari , S. Bhaduri , and S. B. Bhaduri , “Microwave Activated Combustion Synthesis of Bulk Cobalt Silicides,” Journal of Alloys and Compounds 394 (2005): 160–167.

[20] B. Vaidhyanathan , D. K. Agrawal , and R. Roy , “Novel Synthesis of Nitride Powders by Microwave‐Assisted Combustion,” Journal of Materials Research 15 (2000): 974–981.

[21] J. W. Huang , J. Li , and H. Peng , “Microwave Synthesis of Manganese Nitride,” Powder Metallurgy 50 (2007): 137–141.

[22] K. J. Rao , P. A. Ramakrishnan , and R. Gadagkar , “Microwave Preparation of Oxide Bronzes,” Journal of Solid State Chemistry 148 (1999): 100–107.

[23] D. E. Pivonka and J. R. E. Pfield , “Real‐Time In Situ Raman Analysis of Microwave‐Assisted Organic Reactions,” Applied Spectroscopy 58 (2004): 41–46. 10.1366/000370204322729450 14727719

[24] N. E. Leadbeater and J. R. Schmink , “Use of Raman Spectroscopy as a Tool for In Situ Monitoring of Microwave‐Promoted Reactions,” Nature Protocols 3 (2008): 1–7. 10.1038/nprot.2007.453 18193016

[25] J. R. Schmink , J. L. Holcomb , and N. E. Leadbeater , “Testing the Validity of Microwave‐Interfaced, In Situ Raman Spectroscopy as a Tool for Kinetic Studies,” Organic Letters 11 (2009): 365–368. 10.1021/ol802594s 19072323

[26] N. E. Leadbeater , “In Situ Reaction Monitoring of Microwave‐Mediated Reactions Using IR Spectroscopy,” Chemical Communications 46 (2010): 6693–6695. 10.1039/c0cc01921f 20717594

[27] M. V. Gomez and A. D. L. Hoz , “NMR Reaction Monitoring in Flow Synthesis,” Beilstein Journal of Organic Chemistry 13 (2017): 285–300. 10.3762/bjoc.13.31 PMC5331343 28326137

[28] A. G. Whittaker , A. Harrison , G. S. Oakley , I. D. Youngson , R. K. Heenan , and S. M. King , “Preliminary Experiments on Apparatus for in Situ Studies of Microwave‐Driven Reactions by Small Angle Neutron Scattering,” Review of Scientific Instruments 72 (2001): 173–176.

[29] G. A. Tompsett , B. Panzarella , W. C. Conner , et al., “In Situ Small Angle X‐Ray Scattering, Wide Angle X‐Ray Scattering, and Raman Spectroscopy of Microwave Synthesis,” Review of Scientific Instruments 77 (2006): 124101.

[30] B. Panzarella , G. Tompsett , W. C. Conner , and K. Jones , “In Situ SAXS/WAXS of Zeolite Microwave Synthesis: NaY, NaA, and Beta Zeolites,” ChemPhysChem 8 (2007): 357–369. 10.1002/cphc.200600496 17253593

[31] D. S. Wragg , P. J. Byrne , G. Giriat , et al., “In Situ Comparison of Ionothermal Kinetics Under Microwave and Conventional Heating,” Journal of Physical Chemistry C 113 (2009): 20553–20558.

[32] A. Harrison , R. Ibberson , G. Robb , et al., “In Situ Neutron Diffraction Studies of Single Crystals and Powders During Microwave Irradiation,” Faraday Discussions 122 (2002): 363–393. 10.1039/b203379h 12555868

[33] M. M. Günter , C. Korte , G. Brunauer , H. Boysen , M. Lerch , and E. Suard , “In Situ High Temperature Neutron Diffraction Study of Sr/Mg‐Doped Lanthanum Gallate Superionic Conductors Under Microwave Irradiation,” Zeitschrift fur Anorganische und Allgemeine Chemie 631 (2005): 1277–1284.

[34] G. R. Robb , A. Harrison , and A. G. Whittaker , “Temperature‐Resolved, In‐Situ Powder X‐Ray Diffraction of Silver Iodide Under Microwave Irradiation,” PhysChemComm 5 (2002): 135–137.

[35] J. Jamboretz and C. Birkel , “Raman Thermometry for Temperature Assessment of Inorganic Transformations During Microwave Heating,” Journal of Raman Spectroscopy 56 (2024): 49–56.

[36] Ś. Vaucher , J. M. Catala‐Civera , A. Sarua , J. Pomeroy , and M. Kuball , “Phase Selectivity of Microwave Heating Evidenced by Raman Spectroscopy,” Journal of Applied Physics 99 (2006): 113505.

[37] S. Vaucher , M. Stir , K. Ishizaki , J. M. Catala‐Civera , and R. Nicula , “Reactive Synthesis of Ti‐Al Intermetallics During Microwave Heating in an E‐Field Maximum,” Thermochimica Acta 522 (2011): 151–154.

[38] B. García‐Baños , J. J. Reinosa , F. L. Peñaranda‐Foix , J. F. Fernãndez , and J. M. Catalã‐Civera , “Temperature Assessment of Microwave‐Enhanced Heating Processes,” Scientific Reports 9 (2019): 10809. 10.1038/s41598-019-47296-0 PMC6658534 31346250

[39] S. Park , H. Kim , S. Han , et al., “High‐Throughput Synthesis of Mn‐Based Disordered Rock‐Salt Li‐Ion Cathodes With Improved Rate Capability via Rapid Joule‐Heating,” Advanced Energy Materials 15 (2025): 1–12.

[40] A. Urban , A. Abdellahi , S. Dacek , N. Artrith , and G. Ceder , “Electronic‐Structure Origin of Cation Disorder in Transition‐Metal Oxides,” Physical Review Letters 119 (2017): 176402. 10.1103/PhysRevLett.119.176402 29219459

[41] R. J. Clément , Z. Lun , and G. Ceder , “Cation‐Disordered Rocksalt Transition Metal Oxides and Oxyfluorides for High Energy Lithium‐Ion Cathodes,” Energy and Environmental Science 13 (2020): 345–373.

[42] V. C. Wu , E. A. Lawrence , T. Li , et al., “High Energy Density and Micrometer‐Sized $d^{0}$‐Free Disordered Rocksalt Cathodes,” Energy and Environmental Science 18 (2025): 8918–8928.

[43] V. C. Wu , H. A. Evans , R. Giovine , et al., “Rapid and Energy‐Efficient Synthesis of Disordered Rocksalt Cathodes,” Advanced Energy Materials 13 (2023): 2203860.

[44] V. C. Wu , P. Zhong , J. Ong , et al., “The Limited Incorporation and Role of Fluorine in Mn‐Rich Disordered Rocksalt Cathodes,” ACS Energy Letters 9, no. 6 (2024): 3027–3035. 10.1021/acsenergylett.4c01075 PMC11190981 38911531

[45] D. L. Bish and S. A. Howard , “Quantitative Phase Analysis Using the Rietveld Method,” Journal of Applied Crystallography 21 (1988): 86–91.

[46] C. P. Grey and N. Dupré , “NMR Studies of Cathode Materials for Lithium‐Ion Rechargeable Batteries,” Chemical Reviews 104 (2004): 4493–4512. 10.1021/cr020734p 15669160

[47] A. R. Armstrong , N. Dupre , A. J. Paterson , C. P. Grey , and P. G. Bruce , “Combined Neutron Diffraction, NMR, and Electrochemical Investigation of the Layered‐to‐Spinel Transformation in ${\mathrm{LiMnO}}_{2}$ ,” Chemistry of Materials 16 (2004): 3106–3118.

[48] Y. J. Lee and C. P. Grey , “6li Magic‐Angle Spinning (MAS) NMR Study of Electron Correlations, Magnetic Ordering, and Stability of Lithium Manganese(III) Oxides,” Chemistry of Materials 12 (2000): 3871–3878.

[49] A. R. Armstrong , A. J. Paterson , N. Dupré , C. P. Grey , and P. G. Bruce , “Structural Evolution of Layered ${\mathrm{Li}}_{x}{\mathrm{Mn}}_{y}O_{2}$: Combined Neutron, NMR, and Electrochemical Study,” Chemistry of Materials 19 (2007): 1016–1023.

[50] M. Sawamura , S. Kobayakawa , J. Kikkawa , et al., “Nanostructured ${\mathrm{LiMnO}}_{2}$ With ${\mathrm{Li}}_{3}{\mathrm{PO}}_{4}$ Integrated at the Atomic Scale for High‐Energy Electrode Materials With Reversible Anionic Redox,” ACS Central Science 6 (2020): 2326–2338. 10.1021/acscentsci.0c01200 PMC7760474 33376794

[51] R. J. Clément , D. S. Middlemiss , I. D. Seymour , A. J. Ilott , and C. P. Grey , “Insights Into the Nature and Evolution Upon Electrochemical Cycling of Planar Defects in the β‐${\mathrm{NaMnO}}_{2}$ Na‐Ion Battery Cathode: An NMR and First‐Principles Density Functional Theory Approach,” Chemistry of Materials 28 (2016): 8228–8239.

[52] D. S. Middlemiss , A. J. Ilott , R. J. Clément , F. C. Strobridge , and C. P. Grey , “Density Functional Theory‐Based Bond Pathway Decompositions of Hyperfine Shifts: Equipping Solid‐State NMR to Characterize Atomic Environments in Paramagnetic Materials,” Chemistry of Materials 25 (2013): 1723–1734.

[53] J. N. Reimers , E. W. Fuller , E. Rossen , and J. R. Dahn , “Synthesis and Electrochemical Studies of ${\mathrm{LiMnO}}_{2}$ Prepared at Low Temperatures,” Journal of The Electrochemical Society 140 (1993): 3396.

[54] L. Croguennec , P. Deniard , R. Brec , and A. Lecerf , “Nature of the Stacking Faults in Orthorhombic ${\mathrm{LiMnO}}_{2}$ ,” Journal of Materials Chemistry 7 (1997): 511–516.

[55] Y. J. Lee , F. Wang , and C. P. Grey , “ and MAS NMR Studies of Lithium Manganate Cathode Materials,” Journal of the American Chemical Society 120 (1998): 12601–12613.

[56] F. Dogan , J. R. Croy , M. Balasubramanian , et al., “Solid State NMR Studies of ${\mathrm{Li}}_{2}\mathrm{Mn}O_{3}$ and Li‐Rich Cathode Materials: Proton Insertion, Local Structure, and Voltage Fade,” Journal of the Electrochemical Society 162 (2015): A235–A243.

[57] J. Serrano‐Sevillano , D. Carlier , A. Saracibar , J. M. L. D. Amo , and M. Casas‐Cabanas , “DFT‐Assisted Solid‐State NMR Characterization of Defects in ${\mathrm{Li}}_{2}\mathrm{Mn}O_{3}$ ,” Inorganic Chemistry 58 (2019): 8347–8356. 10.1021/acs.inorgchem.9b00394 31199135

[58] R. Giovine , E. Yoshida , V. C. Wu , et al., “An Experimental Approach to Assess Fluorine Incorporation Into Disordered Rock Salt Oxide Cathodes,” Chemistry of Materials 36 (2024): 3643–3654. 10.1021/acs.chemmater.3c03138 PMC11044264 38681087

[59] W. D. Richards , S. T. Dacek , D. A. Kitchaev , and G. Ceder , “Fluorination of Lithium‐Excess Transition Metal Oxide Cathode Materials,” Advanced Energy Materials 8 (2018): 1701533.

[60] R. L. Kam , S. Wang , and G. Ceder , “Thermodynamic Accessibility of Li‐Mn‐Ti‐O Cation Disordered Rock‐Salt Phases,” Journal of Materials Chemistry A (2026), 10.1039/d6ta03748h.

[61] G. Ceder and S. K. Mishra , “The Stability of Orthorhombic and Monoclinic‐Layered ${\mathrm{LiMnO}}_{2}$ ,” Electrochemical and Solid‐State Letters 2 (1999): 550–552.

[62] R. L. Kam , L. Binci , A. D. Kaplan , K. A. Persson , N. Marzari , and G. Ceder , “Interplay Between Electron Localization, Magnetic Order, and Jahn‐Teller Distortion Dictates ${\mathrm{LiMnO}}_{2}$ Phase Stability,” Physical Review B 111 (2025): 245132.

[63] H. M. Hau , T. Mishra , C. Ophus , et al., “Earth‐Abundant Li‐Ion Cathode Materials With Nanoengineered Microstructures,” Nature Nanotechnology 19 (2024): 1831–1839. 10.1038/s41565-024-01787-y PMC11638071 39300225

[64] J. Ahn , R. Giovine , V. C. Wu , et al., “Ultrahigh‐Capacity Rocksalt Cathodes Enabled by Cycling‐Activated Structural Changes,” Advanced Energy Materials 13 (2023): 2300221.

[65] T. Li , T. S. Geraci , K. P. Koirala , et al., “Structural Evolution in Disordered Rock Salt Cathodes,” Journal of the American Chemical Society 146 (2024): 24296–24309. 10.1021/jacs.4c04639 PMC11378274 39172075

[66] T. Li , O. Marques , T. S. Geraci , et al., “Impact of the $d^{0}$ Transition Metal on Local Structural Transformations in Disordered Rock Salt Cathodes,” Chemical Science 17 (2026): 634–651. 10.1039/d5sc06959a PMC12606878 41235074

[67] Z. Cai , B. Ouyang , H.‐M. Hau , et al., “In Situ Formed Partially Disordered Phases as Earth‐Abundant Mn‐Rich Cathode Materials,” Nature Energy 9 (2023): 27–36.

[68] T. Holstun , T. P. Mishra , L. Huang , et al., “Accelerating the Electrochemical Formation of the δ Phase in Manganese‐Rich Rocksalt Cathodes,” Advanced Materials 37 (2024): 2412871. 10.1002/adma.202412871 PMC11817910 39711222

[69] R. J. Gummow and M. M. Thackeray , “An Investigation of Spinel‐Related and Orthorhombic ${\mathrm{LiMnO}}_{2}$ Cathodes for Rechargeable Lithium Batteries,” Journal of the Electrochemical Society 141 (1994): 1178–1182.

[70] Y. Miyaoka , T. Sato , Y. Oguro , et al., “A Practical and Sustainable Ni/Co‐Free High‐Energy Electrode Material: Nanostructured ${\mathrm{LiMnO}}_{2}$ ,” ACS Central Science 10 (2024): 1718–1732. 10.1021/acscentsci.4c00578 PMC11428382 39345817

[71] Z. Liang , O. Marques , O. A. P. Mellone , et al., “Reaction Mechanism of the Synthesis of a Disordered Rock Salt Cathode Material,” Chemistry of Materials 37 (2025): 3950–3958.

[72] R. David , A. Pautrat , D. Filimonov , et al., “Across the Structural re‐entrant Transition in ${\mathrm{BaFe}}_{2}{\left({\mathrm{PO}}_{4}\right)}_{2}$: Influence of the Two‐Dimensional Ferromagnetism,” Journal of the American Chemical Society 135 (2013): 13023–13029. 10.1021/ja404697b 23968376

[73] A. A. Belik , Y. Matsushita , and D. D. Khalyavin , “Reentrant Structural Transitions and Collapse of Charge and Orbital Orders in Quadruple Perovskites,” Angewandte Chemie 129 (2017): 10559–10563; *Angewandte Chemie International Edition* 56 (2017): 10423–10427. 10.1002/anie.201704798 28670864

[74] A. Y. Chufarov , A. P. Tyutyunnik , N. V. Tarakina , et al., “Thermally‐Induced Reentrant Order‐Disorder‐Order Transitions in $A{\mathrm{La}}_{9}{\left({\mathrm{GeO}}_{4}\right)}_{6}O_{2}$ Apatites,” Advanced Optical Materials 12 (2024): 2401520.

[75] S. A. Redfern , R. J. Harrison , H. O. Stc , and D. R. Wood , “Thermodynamics and Kinetics of Cation Ordering in ${\mathrm{MgAl}}_{2}O_{4}$ Spinel up to from In Situ Neutron Diffraction,” American Mineralogist 84 (1999): 299–310.

[76] H. M. Hau , T. Holstun , E. Lee , et al., “Disordered Rocksalts as High‐Energy and Earth‐Abundant Li‐Ion Cathodes,” Advanced Materials 37 (2025): 2502766. 10.1002/adma.202502766 PMC12631525 40326162

[77] H. Ahmed , M. Woo , N. Dumaresq , et al., “Nucleation‐Promoting and Growth‐Limiting Synthesis of Disordered Rock‐Salt Li‐Ion Cathode Materials,” Nature Communications 16 (2025): 5806. 10.1038/s41467-025-60946-4 PMC12216312 40593730

[78] H. Wang , Y. Jang , and Y. Chiang , “Origin of Cycling Stability in Monoclinic‐ and Orthorhombic‐Phase Lithium Manganese Oxide Cathodes,” Electrochemical and Solid‐State Letters 2 (1999): 490–493.

[79] Y. M. Chiang , H. Wang , and Y. I. Jang , “Electrochemically Induced Cation Disorder and Phase Transformations in Lithium Intercalation Oxides,” Chemistry of Materials 13 (2001): 53–63.

[80] Y. Jang , B. Huang , H. Wang , D. Sadoway , and Y. Chiang , “Electrochemical Cycling‐Induced Spinel Formation in High‐Charge‐Capacity Orthorhombic ${\mathrm{LiMnO}}_{2}$ ,” Journal of the Electrochemical Society 146 (1999): 3217–3223.

