# Supplementary material for: Monitoring the Microwave Synthesis of d 0‐Free Disordered Rocksalt Cathodes Using In Situ Infrared Pyrometry
Source: Angew Chem Int Ed Engl. 2026 Aug 5;65(40):e1884534. doi: 10.1002/anie.1884534 (PMC13618298; doi:10.1002/anie.1884534)
Supplement: Supplementary file 1 [file ANIE-65-e1884534-s001.pdf]

Supplementary Information for: Monitoring the microwave synthesis of d^0-free disordered rocksalt cathodes using *in situ* infrared pyrometry

Erick A. Lawrence,[†] Matthew A. Wright,[†] Tianyu Li,[†,‡] Euan N. Bassey,[†,¶] Vijay Kumar,[§] Shiyu Yuan,[†] Yangying Zhu,[§] Pierre-Etienne Cabelguen,[‖] and Raphaële J. Clément[*,†]

†*Materials Department and Materials Research Laboratory, University of California Santa Barbara, Santa Barbara, CA, 93106 USA*

‡*Present address: Department of Chemistry, Louisiana Tech University, Ruston, LA, 71272 USA*

¶*Present address: Centre de RMN à Très Hauts Champs de Lyon (UMR 5082 CNRS, ENS Lyon, UCB Lyon 1), Universite de Lyon, 69100 Villeurbanne, France*

§*Department of Mechanical Engineering, University of California Santa Barbara, Santa Barbara, CA, 93106 USA*

‖*Umicore, New Business Incubation, 31 rue du Marais, Brussels, 1000 Belgium*

E-mail: rclement@ucsb.edu

Experimental

Synthesis

Stoichiometric amounts of Li_2CO_3, LiF, $Mn(III)_2O_3$, $Mn(IV)O_2$ were used to prepare $Li_{1.1}$ $Mn(III)_{0.8}Mn(IV)_{0.1}O_{1.9}F_{0.1}$ (LM9). Precursors were mixed by mechanochemical milling with ethanol at 300 rpm, dried, and then pressed into 200 mg pellets. A double crucible setup was used for microwave synthesis, where a small alumina crucible was placed inside a larger crucible filled with 5 g of activated charcoal. The precursor pellet was placed in the small alumina crucible on top of a layer of sacrificial powder to prevent reaction between the alumina surface and the pellet. The entire setup was placed in a Milestone Pyro Advanced Microwave Muffle reactor and heated at various power levels for various times in ambient air. *In situ* temperature analysis used an Optris PI 1M infrared pyrometer camera (temperature range from 525 to 1500 °C). The emissivity is assumed to be constant as 1. The assumption of constant emissivity even at temperature exceeding 1000 °C was validated by comparing IR camera temperature measurements with spectropyrometer (FAR Associates, Model: FMP2C) temperature readings for same experimental conditions (Supplemental Note 2).

After heating, the pellet was immediately quenched in a beaker of distilled water. The DRX pellet was then dried on a hot plate and ground to a fine powder for further characterization and electrochemistry.

Characterization

Synchrotron powder x-ray diffraction data was collected on high-resolution beamline I11 at the Diamond Light Source.[1,2] The patterns were recorded in the transmission mode ($2.1° < 2\theta < 69.2°$) using a position sensitive detector across three sessions (PSD, λ=0.824297, 0.824640, and 0.826516 Å) at room temperature on samples introduced into 0.5 mm diameter borosilicate glass capillaries. The TOPAS software suite was used for Rietveld refinements of diffraction patterns.[3] The background was fitted using a Chebyshev polynomial function containing 12 coefficients for all samples other than 1.5–2.5 minutes which instead required backgrounds to be fit manually using 36-part linear interpolation. Fundamental parameters of the beamline were modeled, including: Lorentz-polarization, l_a, l_o, and l_h Voigt coefficients, source-to-sample radius, and slit widths. Peak profiles were fitted for all phases using a Pseudo-Voigt model with the exception of monoclinic m−$LiMnO_2$ which was fitted using a Stephens model in the monoclinic setting (b-axis unique). A simple axial model was incorporated and initially constrained to 5.0 mm before being refined after initial peak profile fitting.

X-ray absorption spectroscopy was performed on beamline 4-1 at SSRL. The ATHENA suite was used for all XAS analysis.[4]

Scanning electron microscopy images were obtained using a Thermo Fisher Apreo C Lo-Vac SEM instrument with an accelerating voltage of 5 keV and current of 0.4 nA. Micrograph analysis for particle size distributions was performed by measuring particles in ImageJ, and the reported particle size metrics result from >1000 individual particles per sample.

Elemental analysis was performed using inductively-coupled plasma optical emission spectroscopy (ICP-OES, Agilent 5800 ICP-OES). DRX samples were digested in a 4:1 mixture

of nitric acid and hydrochloric acid before being diluted with distilled water. ICP standards were purchased from Texas Scientific Products.

Solid state ^{7}Li NMR spectra were acquired on a 2.35 T magnet (corresponding to 100 MHz for ^{1}H) using a wide bore Bruker BioSpin spectrometer equipped with a DMX 500 MHz console and a custom-made 1.3 mm single X-broadband magic angle spinning (MAS) probe tuned to ^{7}Li (38.9 MHz). Magic-angle spinning speeds of 60 kHz were used for all samples. Samples were packed into 1.3 mm zirconia rotors. *Ex-situ* cathode samples were center-packed with PTFE tape. ^{7}Li chemical shifts were externally referenced using 1.0 M aqueous LiCl (^{7}Li $\delta_{iso} = 0$ ppm). To improve the signal-to-noise ratio, 40960 transients were averaged using a rotor-synchronized spin-echo pulse sequences with a recycle delay of 20 ms. Quantitative spectra were gathered by averaging 2048 transients with a much longer 10 s recycle delay to fully capture the diamagnetic contributions. Solid-state NMR data were processed using Bruker TopSpin 3.6.0 and spectra were fit using DMFIT.[5]

Electrochemistry

The electrochemical performance of the DRX samples was evaluated using coin cells assembled inside an argon-filled glovebox. Electrode films were prepared with a composition ratio of 70:20:10 for active material, Super C65 carbon, and polytetrafluoroethylene (PTFE), respectively. The as-synthesized DRX powders were first dried in a vacuum oven overnight at 80 °C before being hand-ground with Super C65 in a mortar and pestle for 5 minutes. PTFE was then incorporated into the DRX/carbon mixture to form standalone dry cathode films, which were rolled out and punched into 6.35 mm diameter discs with loading densities ranging from 5 to 6 mg/cm^{-2}. CR2023-type coin cells were assembled using the cathode films paired with lithium metal anodes. The electrolyte consisted of 1M $LiPF_6$ in a 1:1 (v/v) mixture of ethylene carbonate (EC) and dimethyl carbonate (DMC), with Whatman GF/D glass fiber used as the separator. Electrochemical pulse formation[6,7] was performed using parameters optimized for micrometer-sized LM9[7] (50 pulses at 100 mA/g in a narrowed voltage range of 3.5 to 4.5 V) at 50 °C in a temperature-controlled chamber (Neware MHW-25-S) using a Biologic VMP-3 testing system. Subsequent cycling was performed using a current density of 20 mA/g and a voltage range of 1.5 to 4.8 V vs Li/Li^+ at 25 °C in a temperature-controlled chamber (Neware MHW-25-S) using a Biologic VMP-3 testing system.

Supporting Figures and Notes

Supplemental Note 1: Impact of microwave synthesis on carbonate formation in DRX materials

Surface carbonate impurity phases in (Li-rich) oxide cathodes have been linked to reduced coulombic efficiency and rate capability,[8] and enhanced reactivity with the electrolyte.[9] In this section, we examine the formation of (amorphous) carbonate species on DRX particles during microwave synthesis.

The major surface carbonate phase is expected to be Li_2CO_3 as $MnCO_3$ is considerably less stable. Carbon dioxide (CO_2) gas may arise from multiple sources during microwave heating, making it difficult to definitively assign the origin of surface Li_2CO_3 species. One contributing source is the charcoal susceptor: based on the measured mass loss of charcoal ($\approx$ 700 mg), we estimate that 2500 mg of CO_2 is produced during a 5-minute microwave synthesis. In addition, Li_2CO_3 is used as a Li precursor, and its decomposition during formation of the initial ternary intermediate phases is expected to release about $\approx$ 40 mg of CO_2 from a 200 mg pellet. The water quench step may also contribute to surface carbonate formation, as dissolved CO_2 in water can react with the particle surface upon contact. Finally, because the synthesis is conducted in air, atmospheric CO_2 may further contribute to surface carbonate species.

To assess the formation of potentially amorphous Li- and F-containing impurity phases, and in particular Li_2CO_3, during microwave synthesis of DRX materials, we turn to ^{7}Li and ^{19}F solid-state NMR. Indeed, while Li/F species in the Mn-containing cathode give rise to broad and highly shifted paramagnetic NMR resonances, Li/F species in diamagnetic phases such as Li_2CO_3, LiOH, or LiF, result in sharp and overlapping resonances near 0 ppm. This clear contrast enables straightforward differentiation between Li/F environments in the DRX framework and those in secondary impurity phases.

We assume hereafter that the only Li-containing diamagnetic impurity phases are LiF and Li_2CO_3, neglecting LiOH and Li_2O, which would further decrease the upper bound of Li_2CO_3 in the sample. In our previous work on microwave-synthesized LM9 using a carbon susceptor,[7] a weak diamagnetic ^{7}Li NMR signal corresponding to $\approx 2.2\%$ of the total ^{7}Li signal intensity was observed; however, ^{7}Li NMR alone cannot distinguish LiF and Li_2CO_3. To deconvolve these contributions, we first establish lower and upper bounds for LiF, which respectively correspond to the upper and lower bounds for Li_2CO_3.

We use ^{19}F NMR to estimate a lower bound for LiF. The diamagnetic signal in the ^{19}F spectrum is attributed exclusively to LiF and accounts for $\approx 55.3\%$ of the total signal intensity.[7] As discussed in our previous work,[10,11] the paramagnetic ^{19}F NMR signal is systematically underestimated as F species directly bonded to Mn produce resonances that are too broad and too short lived to be observed. To estimate the fraction of F in the DRX phase that is not directly bonded to Mn (and therefore visible by NMR), we assume a random distribution of cations and anions within the DRX structure. Following the method developed in our previous study,[11] the probability of forming F environments in the DRX

phase with no nearest-neighbor paramagnetic TM species is given by:

$$P(F, M_1 \neq M_n) = \left(1 - \frac{S_{M_n}}{2}\right)^6 = \left(1 - \frac{0.93}{2}\right)^6 = 0.023 \quad (1)$$

where S_{Mn} is the Mn stoichiometry in the DRX phase, and $\frac{S_{Mn}}{2}$ is the probability of $M1 = Mn$ for a single $M_1{-}F$ bond when the overall cation stoichiometry is 2. Thus, 2.3% of the F in the DRX phase is not bonded to Mn and is detectable by NMR. This allows an adjusted ^{19}F paramagnetic signal intensity, p', for LM9 to be calculated:

$$p' = \frac{p}{P(F, M1 \neq M_n)} = \frac{0.447}{0.023} = 19.44 \quad (2)$$

where p is the observed paramagnetic intensity. The diamagnetic ^{19}F intensity, d, is 0.553 (55.3% of the observable ^{19}F signal intensity). From these quantities, the fraction of F in the LM9 DRX phase can be expressed as:

$$\frac{p'}{p' + d} = 97.2\% \quad (3)$$

We note that this is an overestimate of the F integrated into the LM9 DRX, as the distribution of species within the DRX is likely non-random, with Li-F bonds being energetically favored over Mn-F bonds. This results in an overestimation of the fraction of "NMR-invisible" F species.

The total F content in the LM9 sample, determined from F-ion selective electrode measurements in our previous work is $F_{0.054}$.[7] Assuming that 97.2% of F is incorporated into the DRX phase, the corresponding F stoichiometry in the DRX phase is $F_{0.0525}$, leaving $F_{0.0015}$ attributable to LiF. This corresponds to a lower bound of 15 mmol of LiF per mole of DRX.

The upper bound of the LiF impurity content is determined by assuming that all diamagnetic ^7Li signal arises from LiF. In contrast to ^{19}F NMR, ^7Li NMR is fully quantitative, as Li is not directly bonded to Mn in the DRX phase. Using the fraction of diamagnetic and paramagnetic ^7Li signal intensities (2.2 and 97.8 %, respectively, as discussed earlier) and the Li stoichiometries of LiF and the DRX phase ($Li_{1.07}$ from ref.[7]), we obtain an upper bound of 24 mmol LiF per mole of DRX. This corresponds to a lower bound of 0 mmol of Li_2CO_3 per mole of DRX in the sample.

Taking the difference between the total diamagnetic Li content in the sample (24 mmol Li per mole of DRX) and the lower bound for the LiF phase (15 mmol Li per mole of DRX), the remaining 9 mmol Li provides an upper bound of 4.5 mmol of Li_2CO_3 per mole of DRX. This value is low and comparable to carbonate contents reported from titration experiments on DRX samples prepared by solid-state synthesis.[11] These results suggest that microwave synthesis using a carbon susceptor and a water quench does not significantly increase the carbonate content in the product relative to traditional furnace-based synthesis.

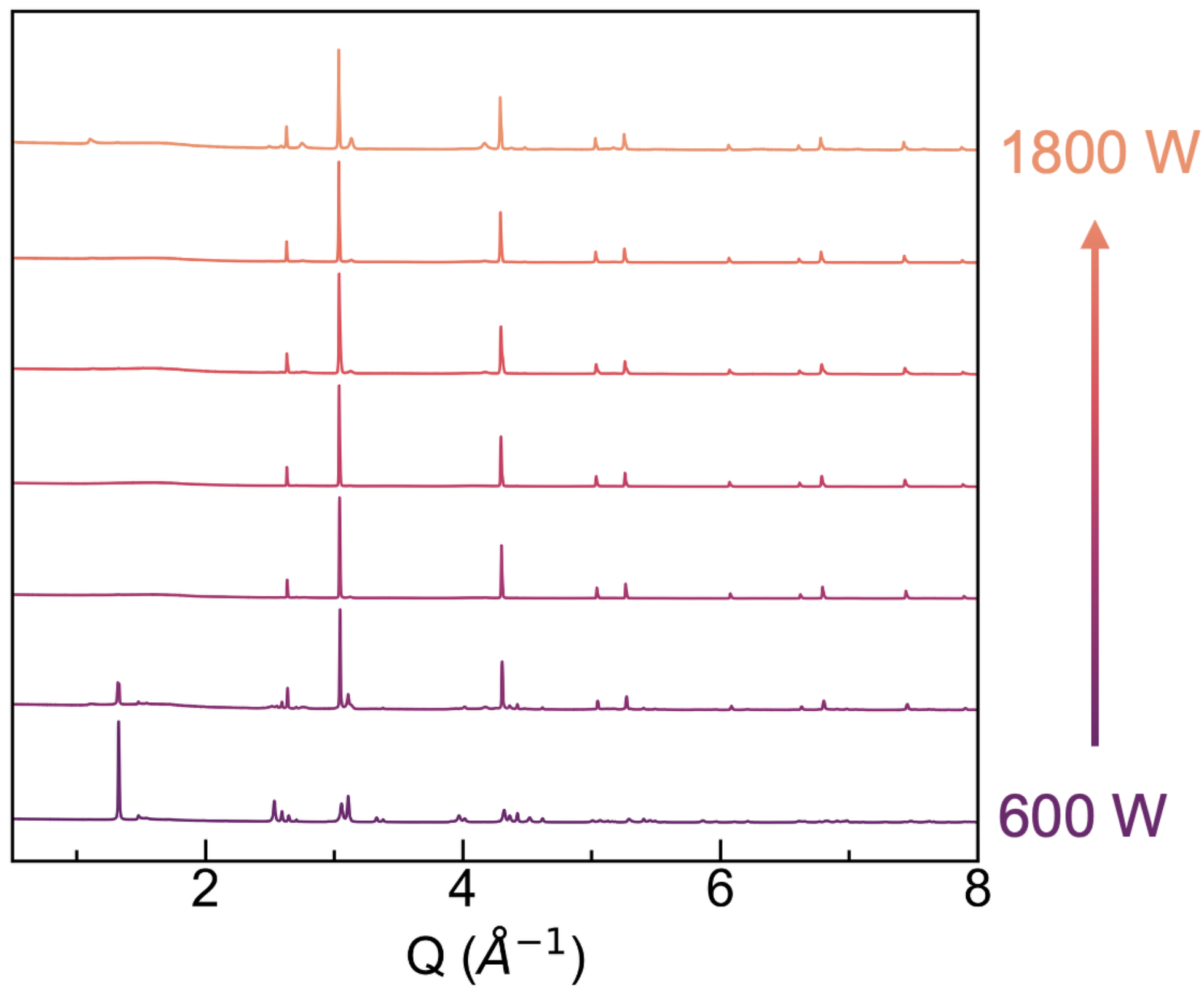

Figure S1: Synchrotron X-ray diffraction (sXRD) patterns of LM9 powder samples quenched after a 5-minute microwave synthesis at applied powers ranging from 600 to 1800 W (in 200 W increments). Samples heated at intermediate power levels (∼1000–1600 W) exhibit comparatively high phase purity.

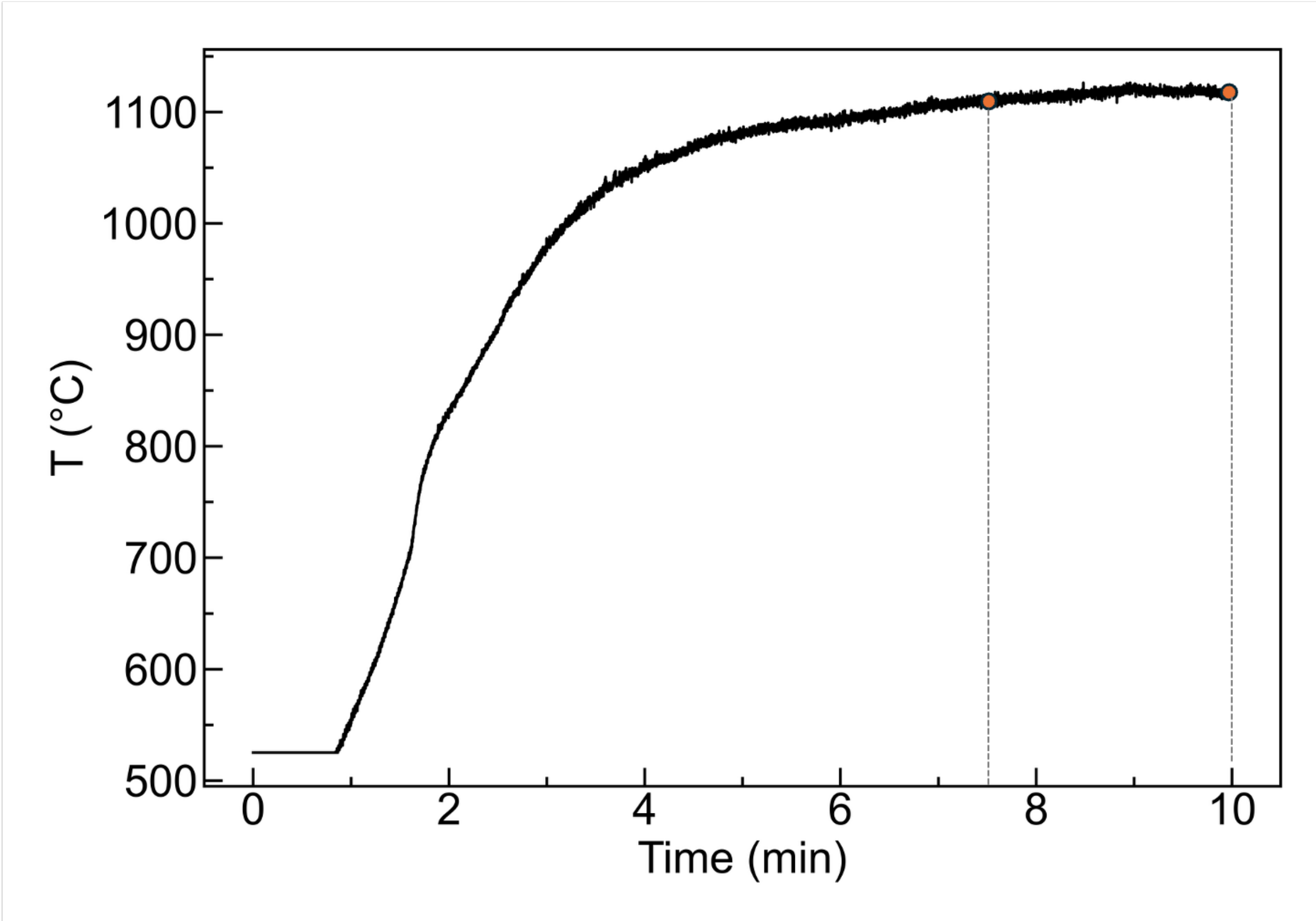

Figure S2: IR thermal profile of LM9 pellet heated for 10 minutes, with orange markers denoting the quench points at 7.5 and 10 minutes.

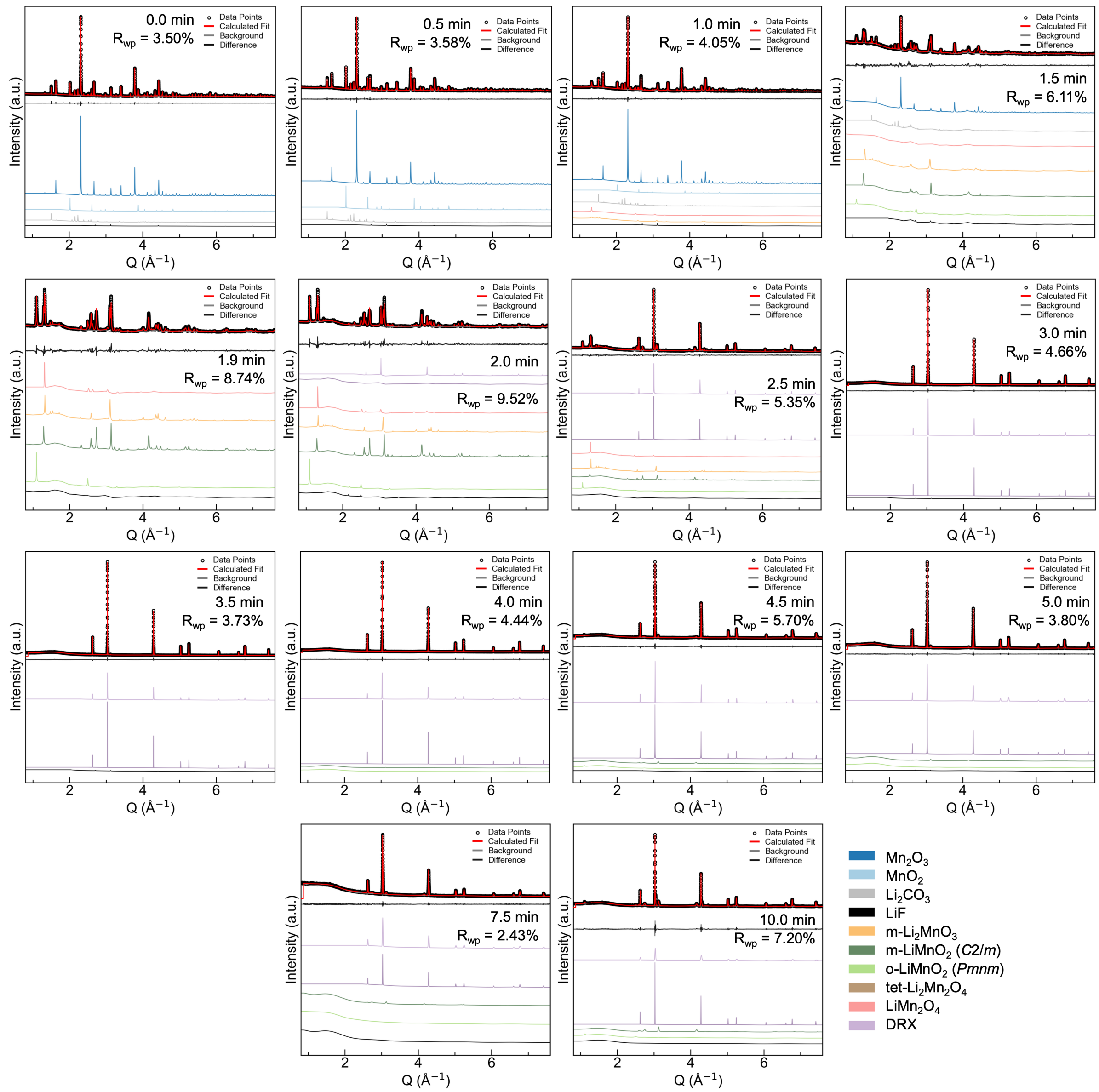

Figure S3: Multiphase Rietveld refinements, including deconvolution of each constituent phase, for samples quenched at different points during the 1200 W microwave synthesis of LM9. Beginning at 2.5 minutes of heating time, the asymmetric DRX peak profiles were modeled using two DRX phases with slightly different lattice parameters starting. The DRX phase fraction reported in the main text corresponds to the sum of the refined fractions of these component DRX phases. The refinement of the layered m$-LiMnO_2$ structure required up to 5 % Li/Mn antisite disorder, consistent with prior reports by Bruce and coworkers.[12]

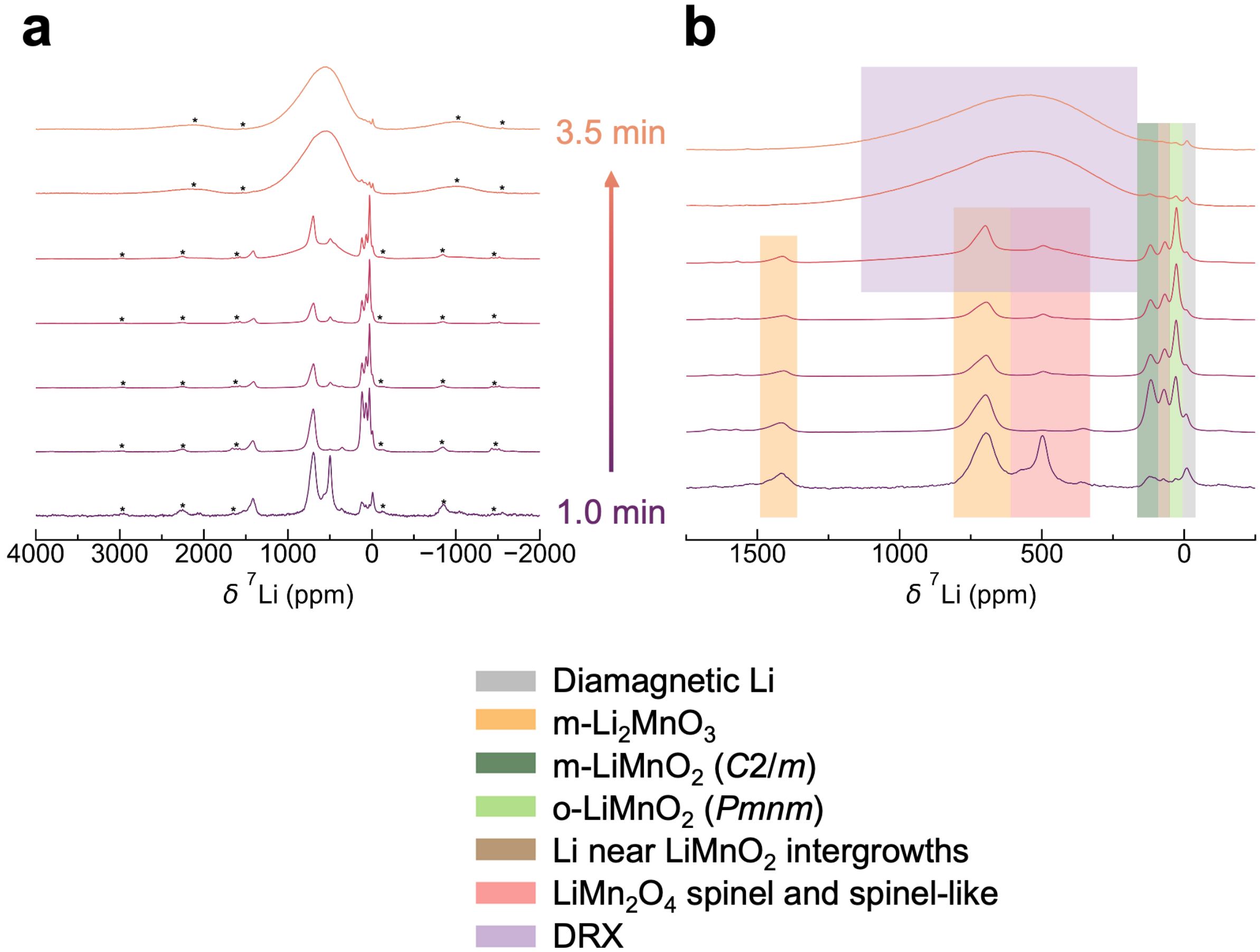

Figure S4: ^{7}Li spin-echo solid-state NMR spectra collected on LM9 powder samples quenched after 1.0 to 3.5 minutes of microwave irradiation at 1200 W. The 1.0 and 3.5 minute limits correspond to the initial appearance of ternary intermediates and the maximum DRX phase purity, respectively. (a) Full spectral width showing both the central resonances and all spinning sidebands (denoted by asterisks). (b) Expanded view of the central resonances only. All spectra were acquired at 2.35 T at a magic angle spinning (MAS) speed of 60 kHz with a 20 ms recycle delay.

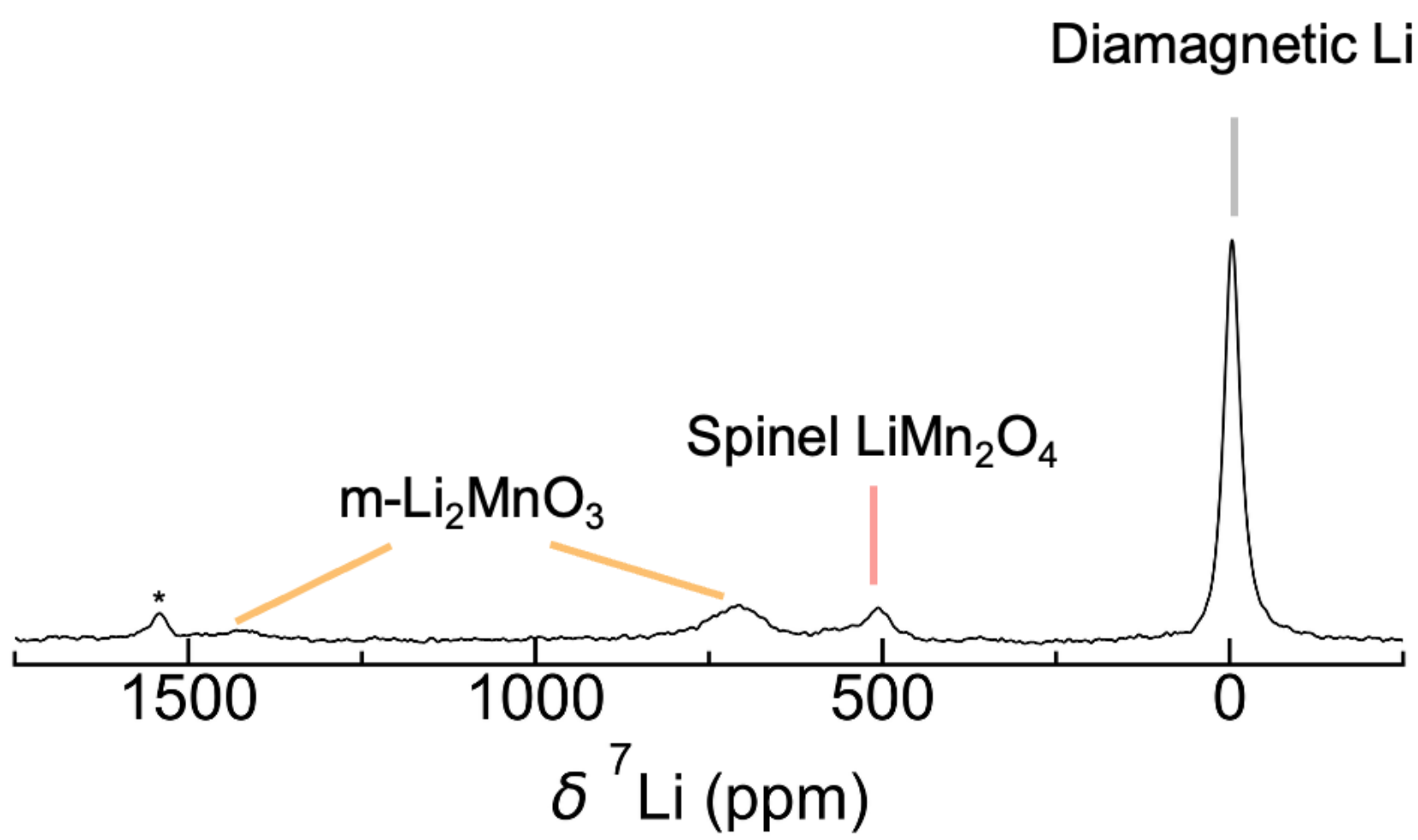

Figure S5: ^{7}Li spin-echo solid-state NMR spectrum collected on an LM9 powder sample quenched after 1.0 minute of microwave irradiation at 1200 W, using a quantitative 10 second recycle delay to ensure full reequilibration of the magnetization of all species between scans. The spectrum was acquired at 2.35 T at a magic angle spinning (MAS) speed of 60 kHz. Spinning sidebands are indicated by asterisks.

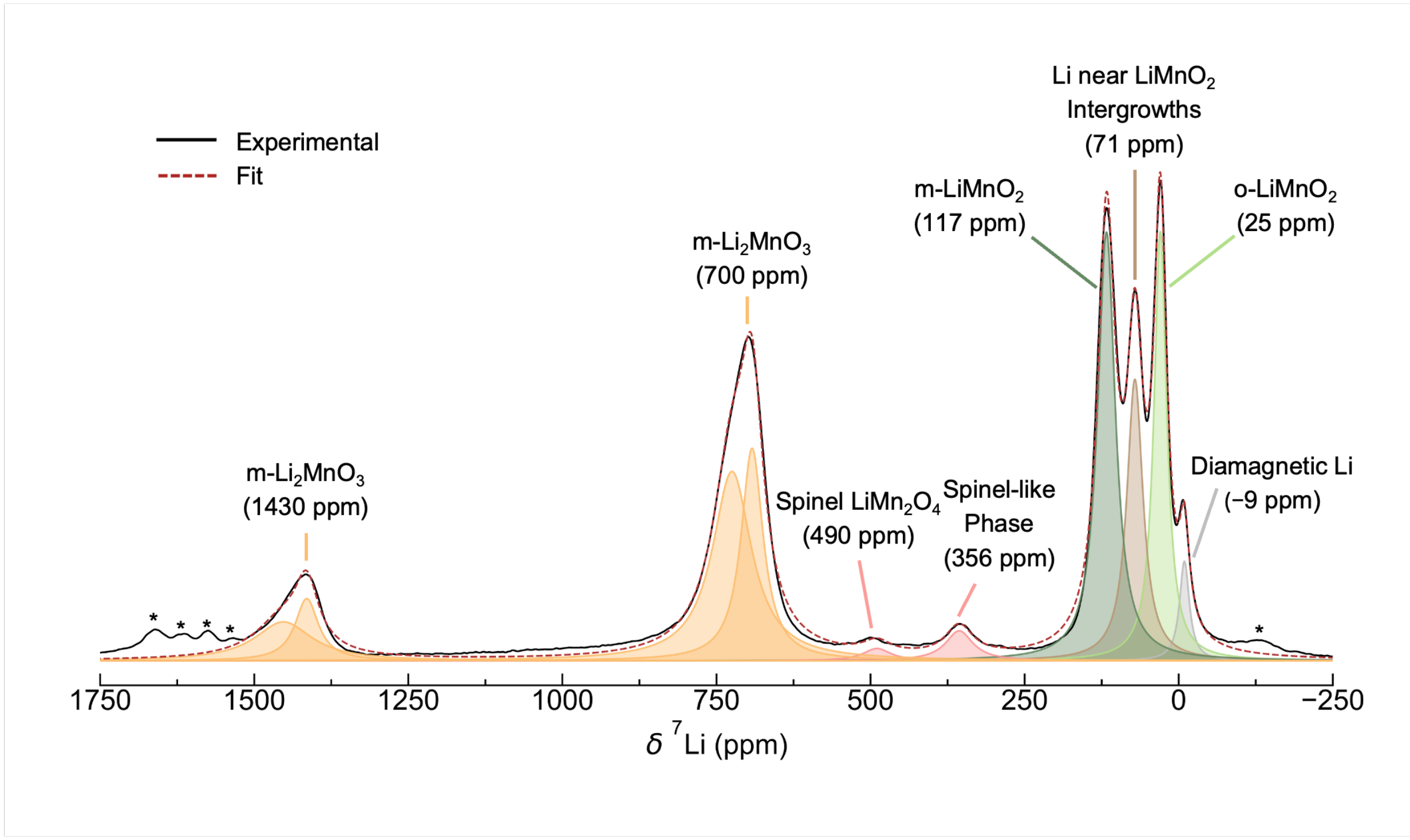

Figure S6: Spectral deconvolution of the ^{7}Li spin-echo solid-state NMR spectrum collected on an LM9 powder sample quenched after 1.5 minutes of microwave irradiation at 1200 W. The spectrum was acquired at 2.35 T at a magic angle spinning (MAS) speed of 60 kHz with a 20 ms recycle delay. Spinning sidebands are indicated by asterisks and were not included in the fit.

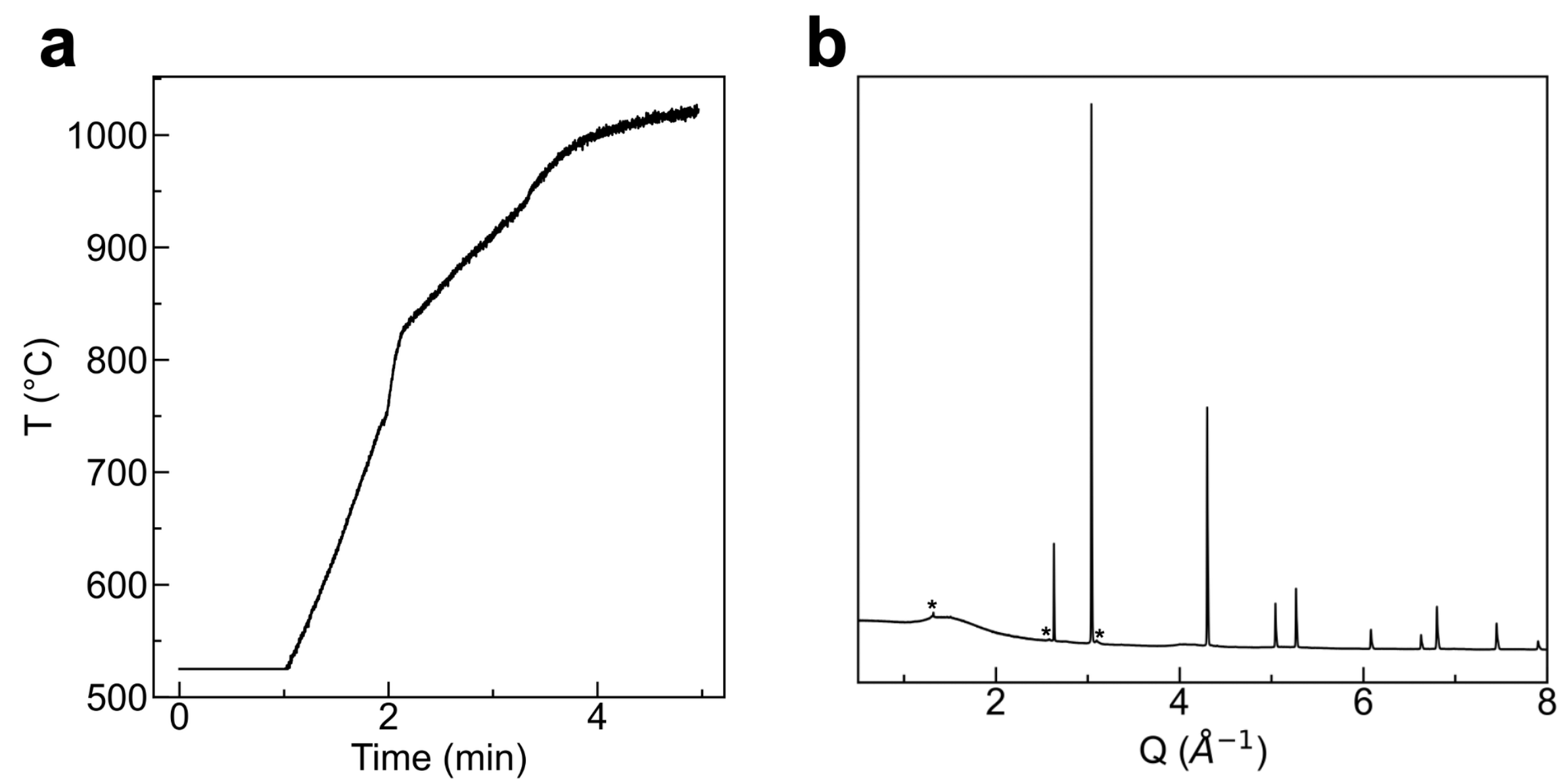

Figure S7: Microwave synthesis of F-free LM9 ($Li_{1.1}Mn_{0.9}O_2$) at 1200 W using identical experimental parameters as for regular (F-containing) LM9. (a) IR thermal profile and (b) sXRD pattern collected on the powder product. We note that small quantities of ordered impurities persist (impurity reflections marked with an asterisk).

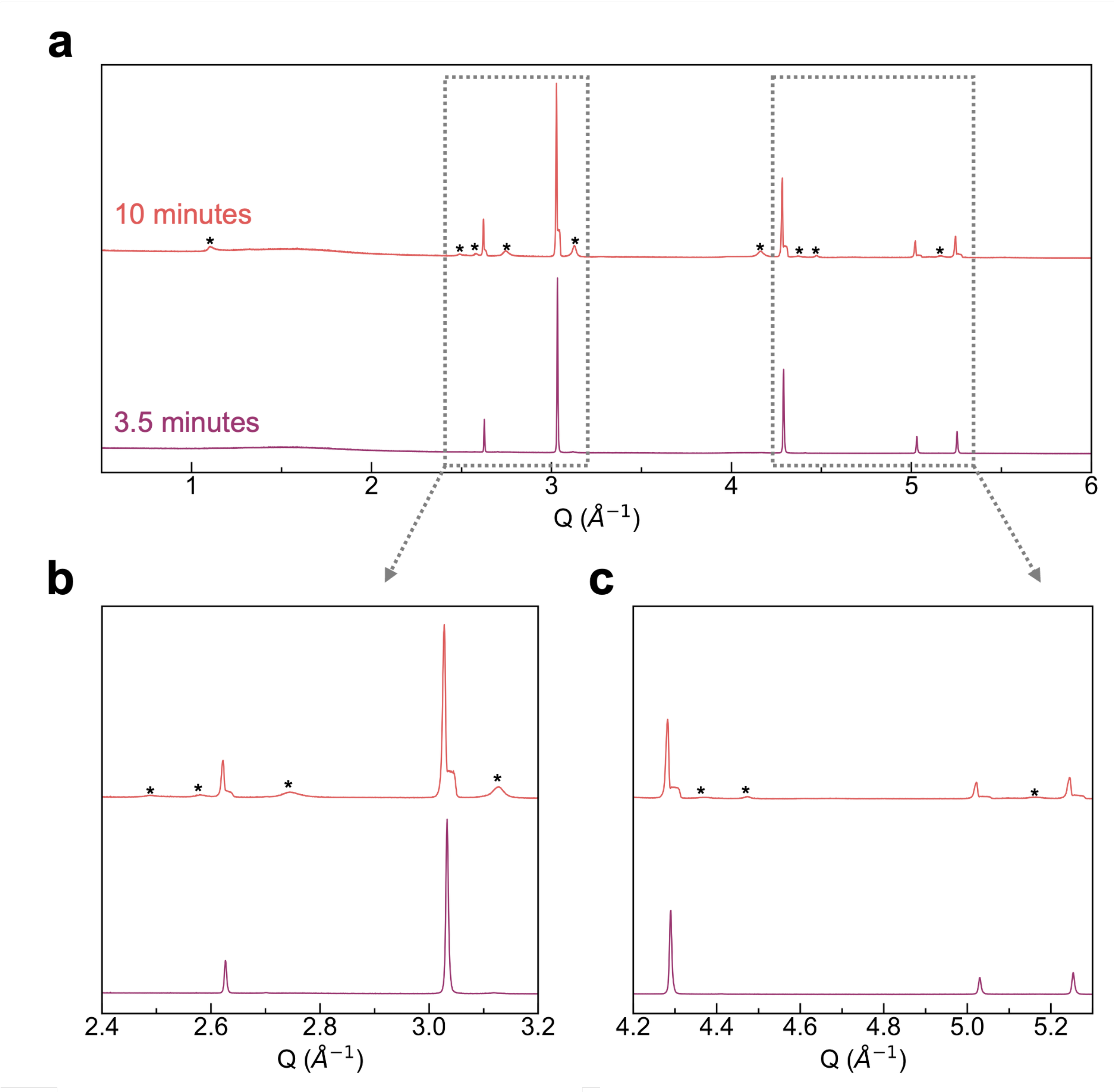

Figure S8: Comparison of the high-Q flat shoulder observed on the DRX peaks in the sample heated for 10 minutes with the phase-pure DRX obtained after 3.5 minutes. Subplots **b** and **c** present magnified views of the relevant peaks. Reflections associated with $LiMnO_2$ phases are indicated by an asterisk.

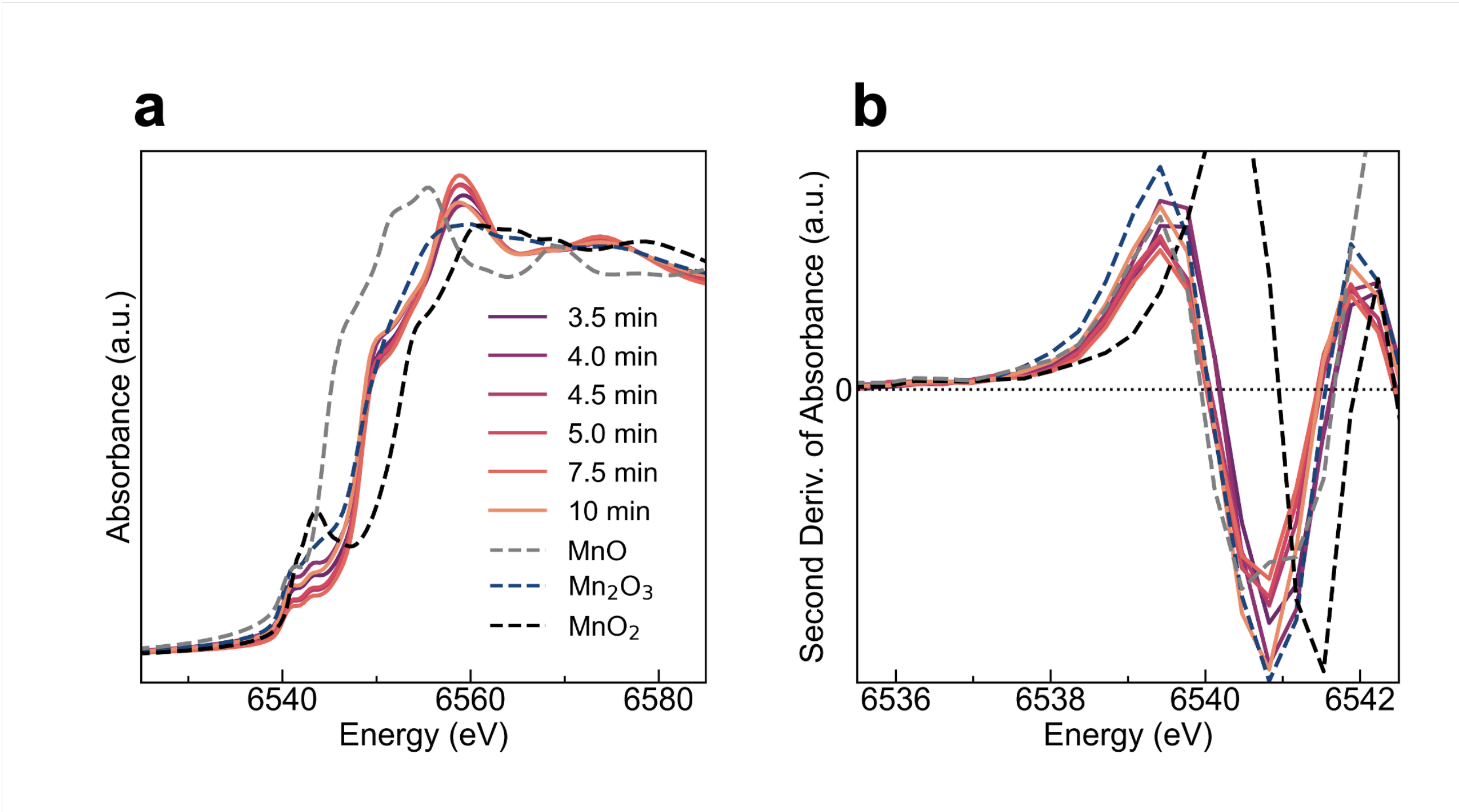

Figure S9: Mn K-edge x-ray absorption (XAS) spectra collected on samples quenched at different points during the 1200 W microwave synthesis of LM9, alongside $Mn^{2+/3+/4+}$ oxide standards shown as dashed reference lines. (a) Near-edge absorption region. (b) Second derivative of the near-edge region to more clearly visualize the onset of the absorption edge. The y-intercept of each trace (highlighted within the dashed box) marks the edge onset; shifts of this intercept to higher or lower energy indicate oxidation or reduction of the average Mn oxidation state, respectively. With increasing synthesis time, the edge onset progressively shifts to lower energy, indicating Mn reduction.

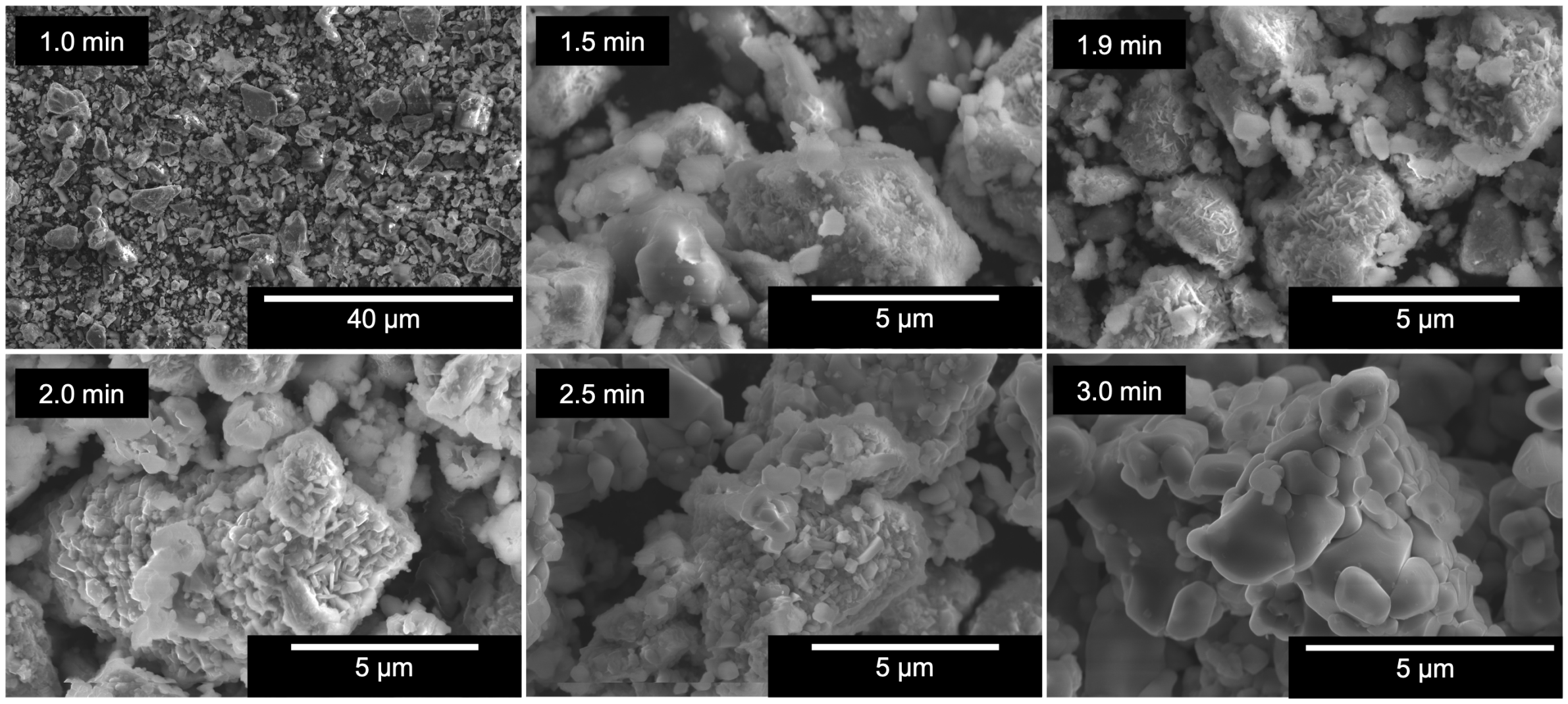

Figure S10: Scanning electron microscope (SEM) micrographs illustrating particle evolution at each quench point during the 1200 W microwave synthesis of LM9. The image of the sample quenched after 1.0 minute was acquired at a lower magnification to highlight the large and broadly distributed precursor particle sizes that persist up to this stage. From the 1.5 minute quench point onward, higher magnification images reveal the emergence of nanoscale surface features, which develop into agglomerated intermediate particles (primary particle size ∼100 nm) before coalescing into micron-sized DRX particles by 3.0 minutes.

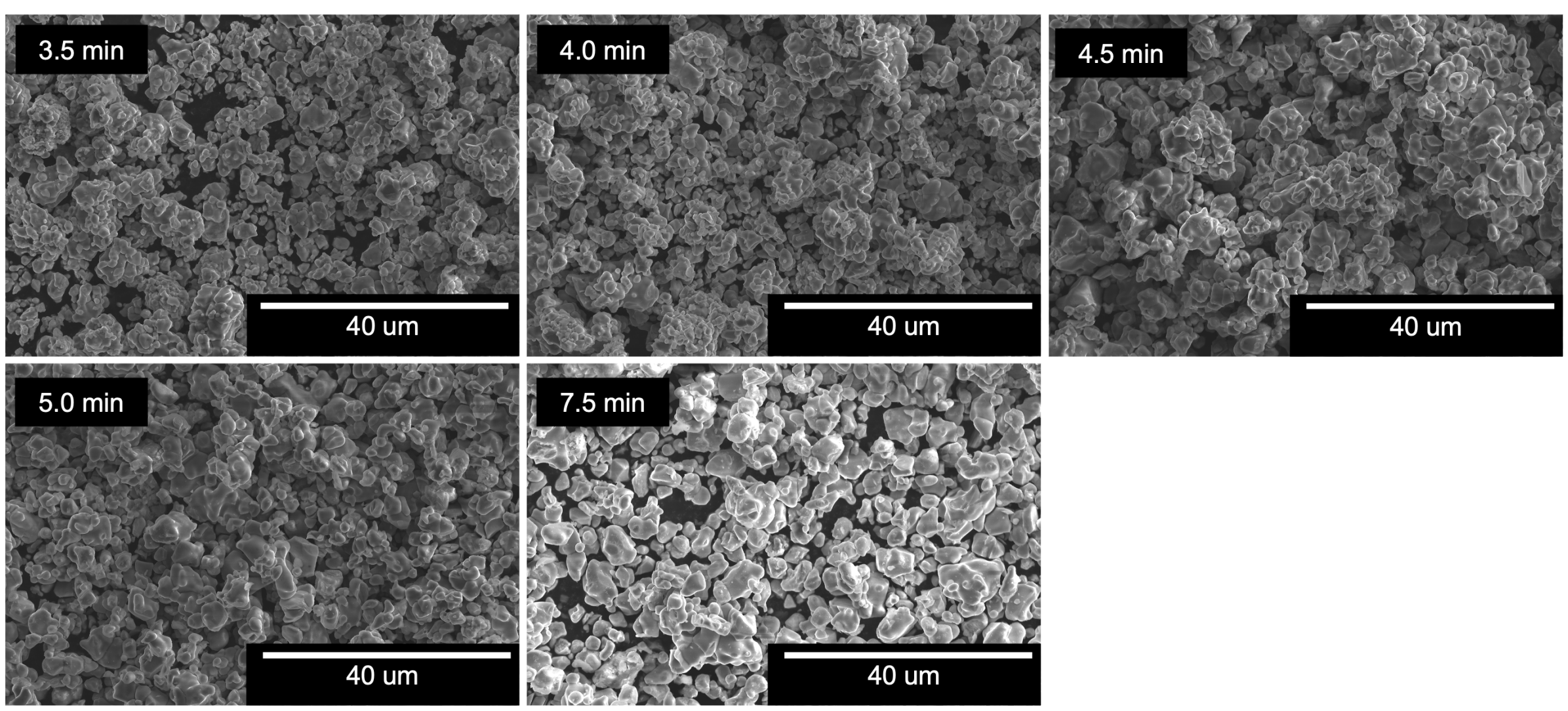

Figure S11: SEM micrographs of LM9 samples quenched after 3.5 to 7.5 minutes, illustrating progressive particle coarsening with increasing heating duration.

Supplemental Note 2: Possible factors affecting the IR temperature profiles

We investigated potential factors affecting the IR temperature-time profiles during LM9 synthesis, including thermal runaway of the crucible or pellet, and temperature-dependent emissivity changes.

First, alumina, which makes up the crucibles, can undergo ”thermal runaway” above ∼900 °C, improving microwave coupling at high temperatures.[13] To test whether this affected heating, we measured the inner crucible floor during synthesis. The thermal profile was smooth and featureless, indicating that alumina did not act as an additional susceptor or cause sharp heating features such as feature 2 in the DRX thermal profiles (Figure S12). These results agree with Kenkre et al., who observed smooth sigmoidal heating of pure alumina without a susceptor.[13]

Second, we examined whether the DRX pellet itself could undergo thermal runaway by coupling directly to the microwave. After a standard 1200 W synthesis, we removed the external crucible and susceptor and then reapplied 1200 W microwave power, comparing this scenario to natural cooling under no power. The schematic workflow and resulting thermal profiles are shown in Figure S13. The hot DRX pellet absorbed enough microwave energy to slow cooling but could not maintain temperature, much less drive heating, indicating that thermal runaway does not occur under these conditions (Figure S13). This contrasts with common assumptions about microwave synthesis of oxides, where exceeding a threshold temperature is expected to trigger thermal runaway and an uncontrolled temperature spike.[14] While metallic precursors can induce thermal runaway, the combination of moderate electronic conduction and microwave absorption, and the small size of LM9 pellets prevents thermal runaway heating under the synthesis conditions used.[15,16]

Third, we evaluated whether temperature-dependent emissivity changes could bias IR camera measurements. Sample emissivity can vary due to factors such as porosity, phase composition, and crystallinity, and it is inherently temperature-dependent. Rapid or large temperature changes during synthesis could therefore potentially distort IR readings. To test the assumption of constant emissivity, we compared IR camera temperatures with those measured using a multi-wavelength spectropyrometer (FAR Associates, Model: FMP2C) under identical conditions for temperatures above 1000 °C. The spectropyrometer accurately determines both temperature and emissivity, though it is limited to temperatures above 1000 °C. Measurements of a DRX pellet after 5 minutes at 1200 W yielded a maximum temperature of 1086 °C, in excellent agreement with the IR camera reading of 1087 °C (assuming an emissivity of 1). These results indicate that emissivity variations have a negligible effect the measured thermal profiles during synthesis.

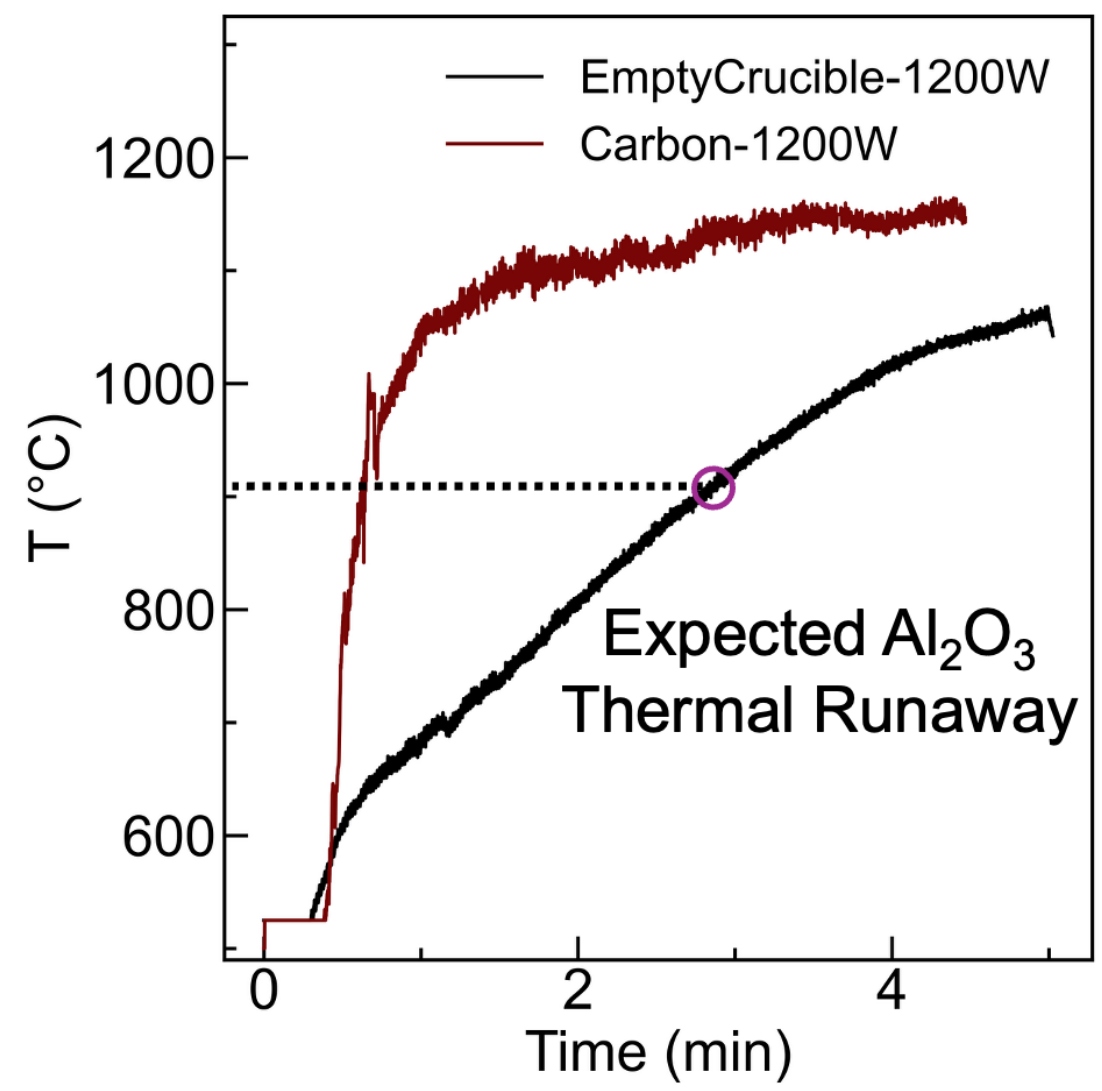

Figure S12: IR heating profile of an empty alumina (Al_2O_3) crucible. Alumina is known to couple directly with microwaves above 900 °C in a process known as ”thermal runaway”.[13] However, the extent of this thermal runaway appears quite weak, and the thermal profile of the empty alumina crucible appears relatively smooth and featureless as it approaches an apparent thermal plateau around 1100 °C.

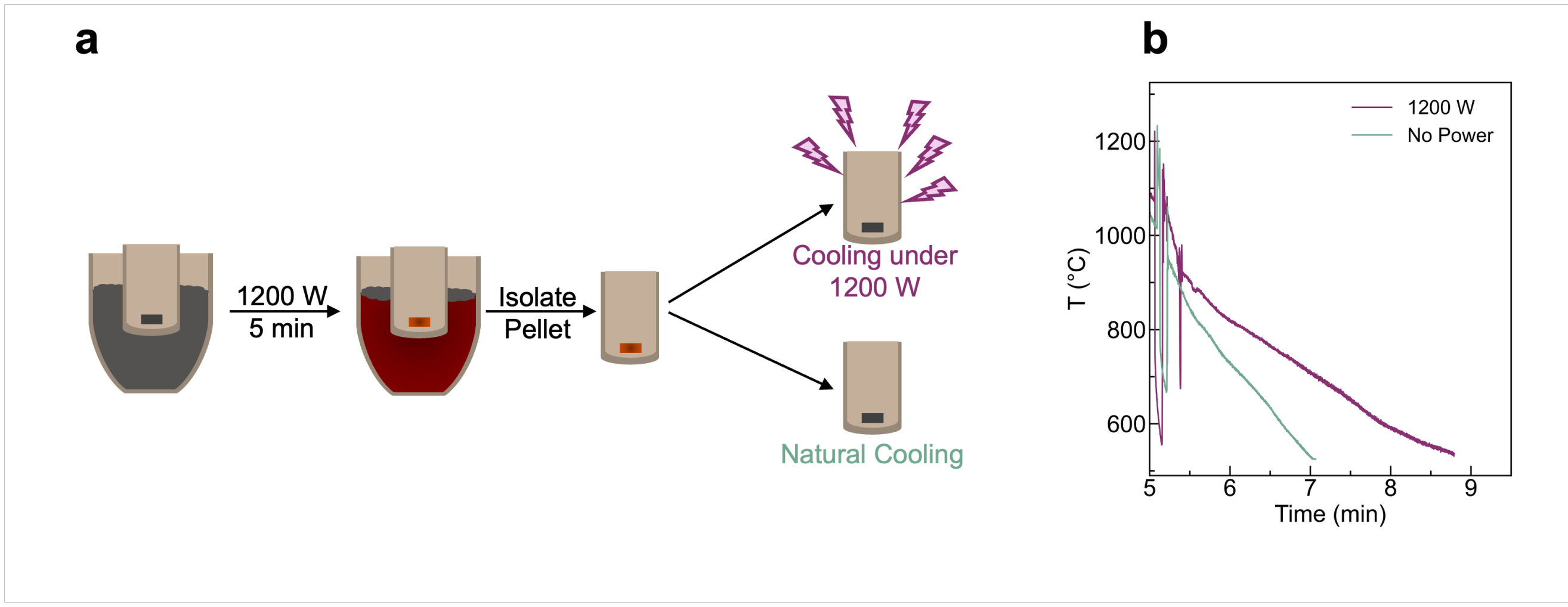

Figure S13: (a) Schematic of the workflow for DRX microwave coupling tests, illustrating how precursor pellets were heated under standard conditions (1200 W, 5 min) using a double-crucible setup with 5 g of activated charcoal susceptor. After heating, the susceptor was removed and the hot internal crucible containing the DRX pellet was returned to the microwave to monitor its cooling profile was observed under (i) no applied microwave power, and (ii) under 1200 W of applied microwave power. (b) Corresponding IR traces of these cooling tests. The first 5 minutes of each trace, corresponding to the standard synthesis heating curve, are omitted. The jagged features between ∼5 and 5.5 minutes reflect the time required to remove the susceptor and reposition the DRX pellet within the IR camera's field of view.

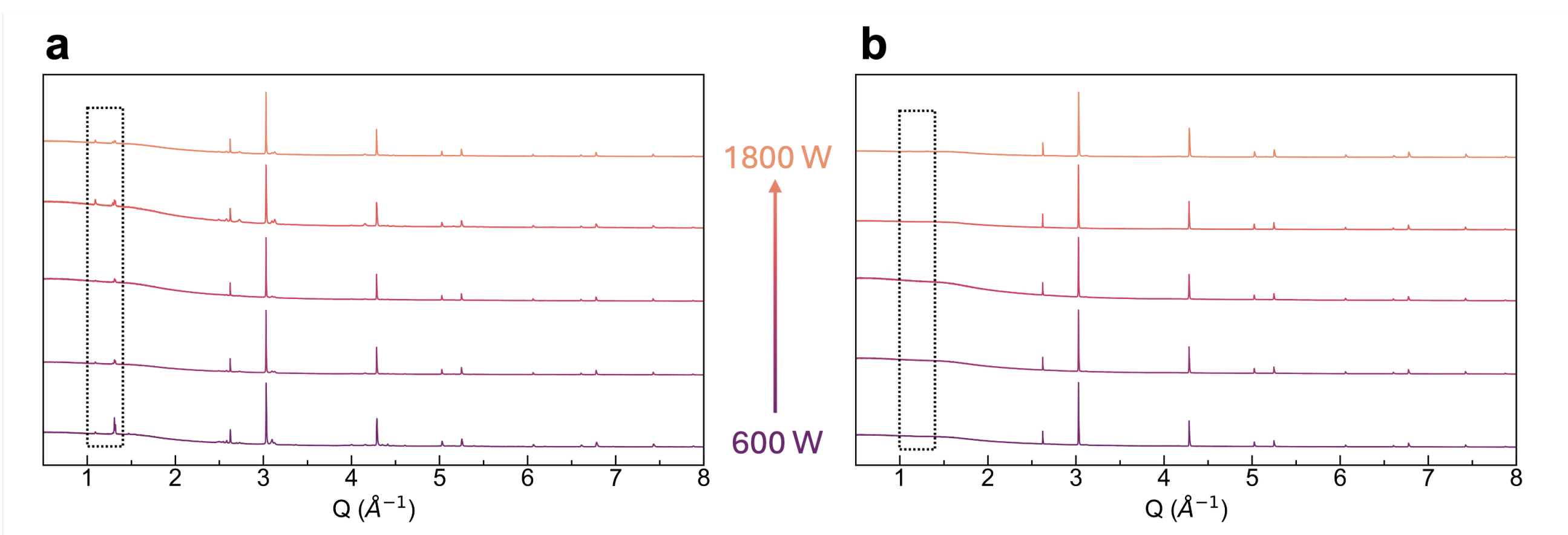

Figure S14: Full sXRD patterns corresponding to the samples shown in Figure 5 of the main text, for LM9 powder samples quenched after heating to (a) 900 °C and (b) 980 °C for varying microwave powers. The dotted boxes indicate the region containing impurity peaks. These impurities present in the 900 °C samples, are absent for the 980 °C samples.

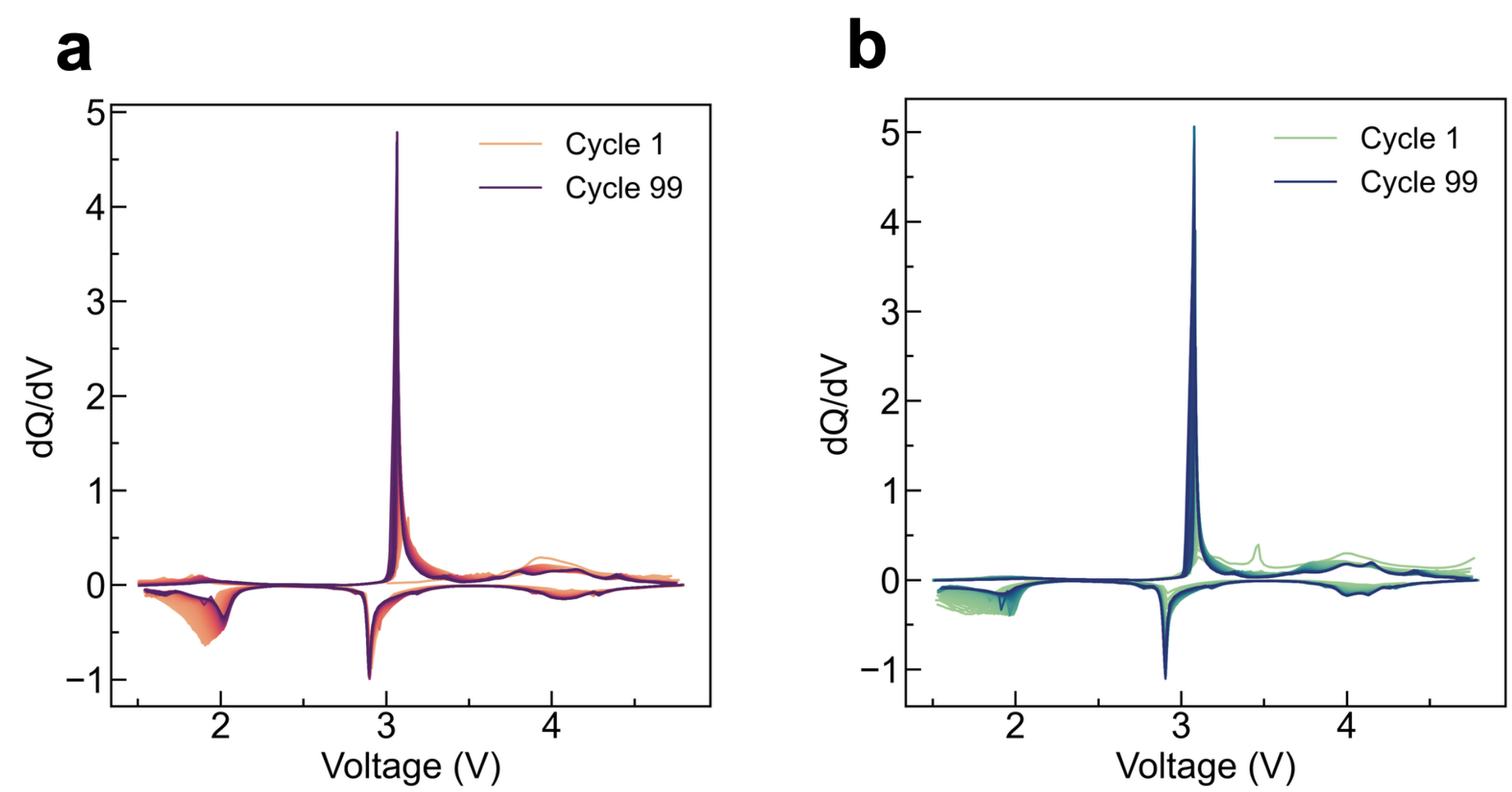

Figure S15: Differential capacity curves of (a) LM9-3.5 and (b) LM9-7.5.

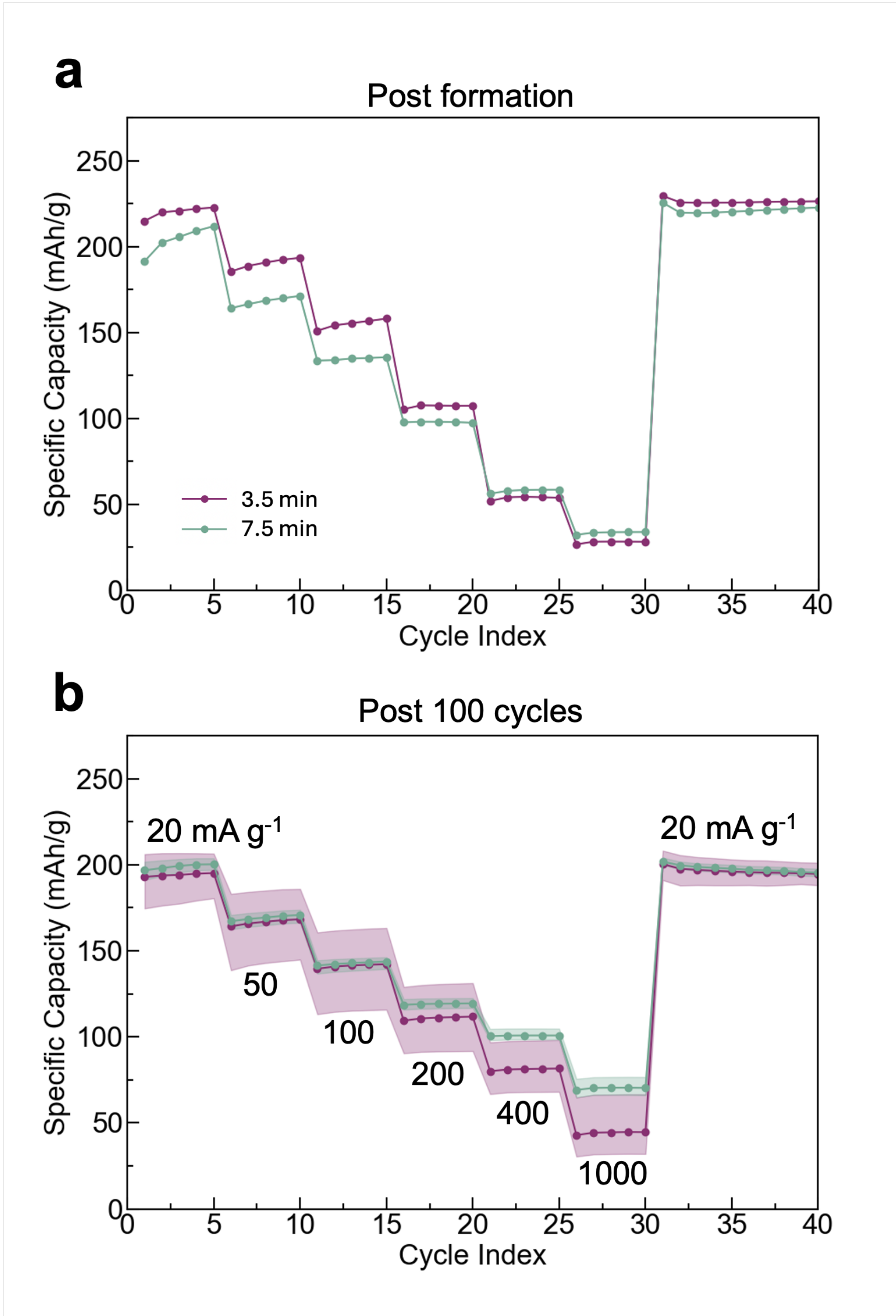

Figure S16: Variable-rate performance of LM9-3.5 min and LM9-7.5 min samples: (a) immediately after pulse formation and (b) after 100 cycles at 20 mA g^{-1}. Due to the incomplete transformation in LM9-7.5, only single cells were tested for post-formation in (a). The cells tested in (b) are those whose 20 mA g^{-1} cycling data is reported in main-text Figure 6.

References

(1) Tartoni, N.; Thompson, S. P.; Tang, C. C.; Willis, B. L.; Derbyshire, G. E.; Wright, A. G.; Jaye, S. C.; Homer, J. M.; Pizzey, J. D.; Bell, A. M. High-performance

X-ray detectors for the new powder diffraction beamline I11 at Diamond. *Journal of Synchrotron Radiation* **2008**, *15*, 43–49.

(2) Thompson, S. P.; Parker, J. E.; Potter, J.; Hill, T. P.; Birt, A.; Cobb, T. M.; Yuan, F.; Tang, C. C. Beamline I11 at Diamond: A new instrument for high resolution powder diffraction. *Review of Scientific Instruments* **2009**, *80*.

(3) Coelho, A. TOPAS and TOPAS-Academic: an optimization program integrating computer algebra and crystallographic objects written in C++. *Journal of Applied Crystallography* **2018**, *51*, 210–218.

(4) Ravel, B.; Newville, M. ATHENA, ARTEMIS, HEPHAESTUS: data analysis for X-ray absorption spectroscopy using IFEFFIT. *Journal of synchrotron radiation* **2005**, *12*, 537–541.

(5) Massiot, D.; Fayon, F.; Capron, M.; King, I.; Calvé, S. L.; Alonso, B.; Durand, J.-O.; Bujoli, B.; Gan, Z.; Hoatson, G. Modelling one- and two-dimensional solid-state NMR spectra. *Magnetic Resonance in Chemistry* **2002**, *40*, 70–76.

(6) Holstun, T.; Mishra, T. P.; Huang, L.; Hau, H. M.; Anand, S.; Yang, X.; Ophus, C.; Bustillo, K.; Ma, L.; Ehrlich, S.; Ceder, G. Accelerating the Electrochemical Formation of the δ Phase in Manganese-Rich Rocksalt Cathodes. *Advanced Materials* **2024**, *37*.

(7) Wu, V. C.; Lawrence, E. A.; Li, T.; Bassey, E. N.; Chang, C. Y.; Hwang, B. J.; Cabelguen, P. E.; Clément, R. J. High energy density and micrometer-sized d0-free disordered rocksalt cathodes. *Energy and Environmental Science* **2025**, *18*, 8918–8928.

(8) Banov, K.; Khodeir, M.; Baster, D.; Kazzi, M. E.; Novák, P. Impact of Trace Impurities in the Electrolyte on the Electrochemistry of Positive Electrodes for Lithium-Ion Batteries. *Journal of The Electrochemical Society* **2025**, *172*, 080515.

(9) Kaufman, L. A.; McCloskey, B. D. Surface Lithium Carbonate Influences Electrolyte Degradation via Reactive Oxygen Attack in Lithium-Excess Cathode Materials. *Chemistry of Materials* **2021**, *33*, 4170–4176.

(10) Clément, R. J.; Kitchaev, D.; Lee, J.; Ceder, G. Short-Range Order and Unusual Modes of Nickel Redox in a Fluorine-Substituted Disordered Rocksalt Oxide Lithium-Ion Cathode. *Chemistry of Materials* **2018**, *30*, 6945–6956.

(11) Giovine, R.; Yoshida, E.; Wu, V. C.; Ji, Y.; Crafton, M. J.; McCloskey, B. D.; Clément, R. J. An Experimental Approach to Assess Fluorine Incorporation into Disordered Rock Salt Oxide Cathodes. *Chemistry of Materials* **2024**, *36*, 3643–3654.

(12) Armstrong, A. R.; Bruce, P. G. Synthesis of layered LiMnO2 as an electrode for rechargeable lithium batteries. *Nature* **1996**, *381*, 499–500.

(13) Kenkre, V. M.; Skala, L.; Weiser, M. W.; Katz, J. D. *Theory of microwave interactions in ceramic materials: the phenomenon of thermal runaway*; 1991; Vol. 26; pp 2483–2489.

(14) Levin, E. E.; Grebenkemper, J. H.; Pollock, T. M.; Seshadri, R. Protocols for High Temperature Assisted-Microwave Preparation of Inorganic Compounds. *Chemistry of Materials* **2019**, *31*, 7151–7159.

(15) Kitchen, H. J.; Vallance, S. R.; Kennedy, J. L.; Tapia-Ruiz, N.; Carassiti, L.; Harrison, A.; Whittaker, A. G.; Drysdale, T. D.; Kingman, S. W.; Gregory, D. H. Modern microwave methods in solid-state inorganic materials chemistry: From fundamentals to manufacturing. *Chemical Reviews* **2014**, *114*, 1170–1206.

(16) Lekse, J. W.; Pischera, A. M.; Aitken, J. A. Understanding solid-state microwave synthesis using the diamond-like semiconductor, AgInSe2, as a case study. *Materials Research Bulletin* **2007**, *42*, 395–403.
